# Intelligent design of polymer nanogels for full-process sensitized radiotherapy and dual-mode computed tomography/magnetic resonance imaging of tumors

**DOI:** 10.7150/thno.70346

**Published:** 2022-04-18

**Authors:** Changchang Zhang, Wenzhi Tu, Xuming Chen, Bing Xu, Xin Li, Chaolei Hu, Mingwu Shen, Shaoli Song, Chunjuan Jiang, Shengyu Yao, Andrij Pich, Yong Liu, Xiangyang Shi

**Affiliations:** 1 State Key Laboratory for Modification of Chemical Fibers and Polymer Materials, Shanghai Engineering Research Center of Nano-Biomaterials and Regenerative Medicine, College of Chemistry, Chemical Engineering and Biotechnology, Donghua University, Shanghai 201620, P. R. China; 2 Department of Radiation Oncology, Shanghai General Hospital, Shanghai Jiao Tong University School of Medicine, Shanghai 200080, P. R. China; 3 DWI-Leibniz-Institute for Interactive Materials e.V., Forckenbeckstr. 50, 52056, Aachen, Germany; 4 Functional and Interactive Polymers, Institute of Technical and Macromolecular Chemistry, RWTH Aachen University, Worringerweg 2, 52074, Aachen, Germany; 5 Department of Nuclear Medicine, Shanghai Cancer Center, Fudan University, Shanghai 200030, P. R. China

**Keywords:** Hybrid PVCL nanogels, Fenton-like reaction, manganese dioxide, tumor radiotherapy, full-process sensitization

## Abstract

**Rationale:** Development of intelligent radiosensitization nanoplatforms for imaging-guided tumor radiotherapy (RT) remains challenging. We report here the construction of an intelligent nanoplatform based on poly(*N*-vinylcaprolactam) (PVCL) nanogels (NGs) co-loaded with gold (Au) and manganese dioxide (MnO_2_) nanoparticles (NPs) for dual-mode computed tomography (CT)/magnetic resonance (MR) imaging-guided “full-process” sensitized RT of tumors.

**Methods:** PVCL NGs were synthesized *via* precipitation polymerization and* in situ* loaded with Au and MnO_2_ NPs. The created PVCL-Au-MnO_2_ NGs were well characterized and systematically examined in their cytotoxicity, cellular uptake, intracellular oxygen and ·OH production, and cell cycle arrest *in vitro*, evaluated to disclose their RT sensitization effects of cancer cells and a tumor model, and assessed to validate their dual-mode CT/MR imaging potential, pharmacokinetics, biodistribution, and biosafety *in vivo*.

**Results:** The formed PVCL-Au-MnO_2_ NGs with a size of 121.5 nm and good stability can efficiently generate reactive oxygen species through a Fenton-like reaction to result in cell cycle distribution toward highly radiosensitive G2/M phase prior to X-ray irradiation, sensitize the RT of cancer cells under X-ray through the loaded Au NPs to induce the significant DNA damage, and further prevent DNA-repairing process after RT through the continuous production of O_2_ catalyzed by MnO_2_ in the hybrid NGs to relieve the tumor hypoxia. Likewise, the *in vivo* tumor RT can also be guided through dual mode CT/MR imaging due to the Au NPs and Mn(II) transformed from MnO_2_ NPs.

**Conclusion:** Our study suggests an intelligent PVCL-based theranostic NG platform that can achieve “full-process” sensitized tumor RT under the guidance of dual-mode CT/MR imaging.

## Introduction

Radiotherapy (RT) has been considered as one of the major cancer treatment modes 1-3. However, owing to the extremely complex tumor microenvironment (TME) featured with hypoxia, slight acidity, overproduction of hydrogen peroxide (H_2_O_2_) and high intracellular concentration of glutathione (GSH), the therapeutic efficacy of RT has been greatly limited. In particular, hypoxia results in radioresistance of cancer cells 3 times higher than the aerobic condition 4, attributing to the mechanism that the DNA radicals formed by ionizing radiation can be easily repaired in the absence of oxygen 5.

To overcome the tumor radioresistance, various strategies have been developed. Among them, one most investigated approach is to develop nanostructured radiosensitizers based on high-Z elements (e.g., Au, Hf, Bi, and Pt, etc.) with enhanced photoelectric and Compton effects to exogenously increase the radiation response of cancer cells to RT 6-9. Additionally, through endogenous modulation of the tumor hypoxia including normalization of tumor vessels 10, 11, intentional delivery of oxygen (O_2_) 12, knockdown of hypoxia-inducible factor-1α (HIF-1α) protein and downstream genes to relieve tumor invasion 13, or catalytic decomposition of endogenous H_2_O_2_ by catalase (CAT) 14, tumor hypoxia can also be relieved for sensitized tumor RT. Though great progresses have been achieved, most of them only focus on a single process during RT (i.e., either by increasing the cellular radiosensitivity prior to ionizing radiation, enhancing the local radiation dose absorption during RT or inhibiting the DNA damage repair after RT), leading to limited radiosensitization efficacy for tumor therapy. For instance, in our previous work, we reported the construction of hypoxia-targeting dendrimer-entrapped Au nanoparticles (NPs) for RT of tumors, where the tumor RT sensitization was only based on the high-Z element Au during RT 15.

In recent years, manganese dioxide (MnO_2_)-based nanomaterials have emerged as promising radiosensitizers for enhanced RT, owing to their excellent TME regulation capacity 16-19. It has been shown that MnO_2_-based nanomaterials display CAT-mimic catalytic activity to decompose H_2_O_2_ in the tumor site to form O_2_, thus alleviating the tumor hypoxia 20-25. Moreover, the MnO_2_ can react with H^+^, H_2_O_2_ and GSH in TME to modulate the TME, thus facilitating enhanced tumor cell apoptosis 26-28. Further, the thus formed Mn^2+^ has been reported to exert chemodynamic therapy (CDT) of tumors through conversion of the endogenous H_2_O_2_ into highly toxic hydroxyl radical (·OH) *via* a Fenton-like reaction to cause the intracellular oxidative stress 29-32. The induced oxidative stress can significantly influence the genetic stability, protein expression and cell cycle distribution, thereby making the cells more susceptible to the further RT treatment 33, 34. Lastly, the generated Mn^2+^ is reported to have a higher r_1_ relaxivity than the commercial Magnevist^@^ for T_1_-weighted magnetic resonance (MR) imaging of tumors 28, 35, 36. Therefore, we hypothesize that by combining the TME regulation capacity of MnO_2_ with the high RT sensitization property of Au NPs, a multiple or “full-process” radiosensitization could be achieved to enhance the RT of tumors.

To realize effective integration of multiple components within a nanosystem, it is desirable to develop a unique carrier system allowing for easy or *in-situ* incorporation of different components with controllable sizes, good colloidal stability and excellent biocompatibility 37-39. Nanogels (NGs), possessing physically or chemically crosslinked 3D networks, have been known as a promising colloidal container for the integration of NPs, as they can be designed to own excellent colloidal stability and compatibility to biological systems 40-42. For instance, we have proven that MnO_2_ NPs can be *in-situ* loaded within poly(*N*-vinylcaprolactam) (PVCL) NGs with a controlled size 27, and conducting polymeric NPs are able to be *in-situ* loaded within poly(γ-glutamic acid) NGs 43, 44 or hyaluronic acid NGs 45. The major advantages of the *in-situ* incorporation method are the controllability of NP size and density in the matrix, the prevention of NP leakage, and the flexibility of NP types. These studies suggest that polymeric NGs may be employed as a unique nanoreactor to *in situ* incorporate single- or multiple-component inorganic NPs for different biomedical applications.

**Scheme 1:**
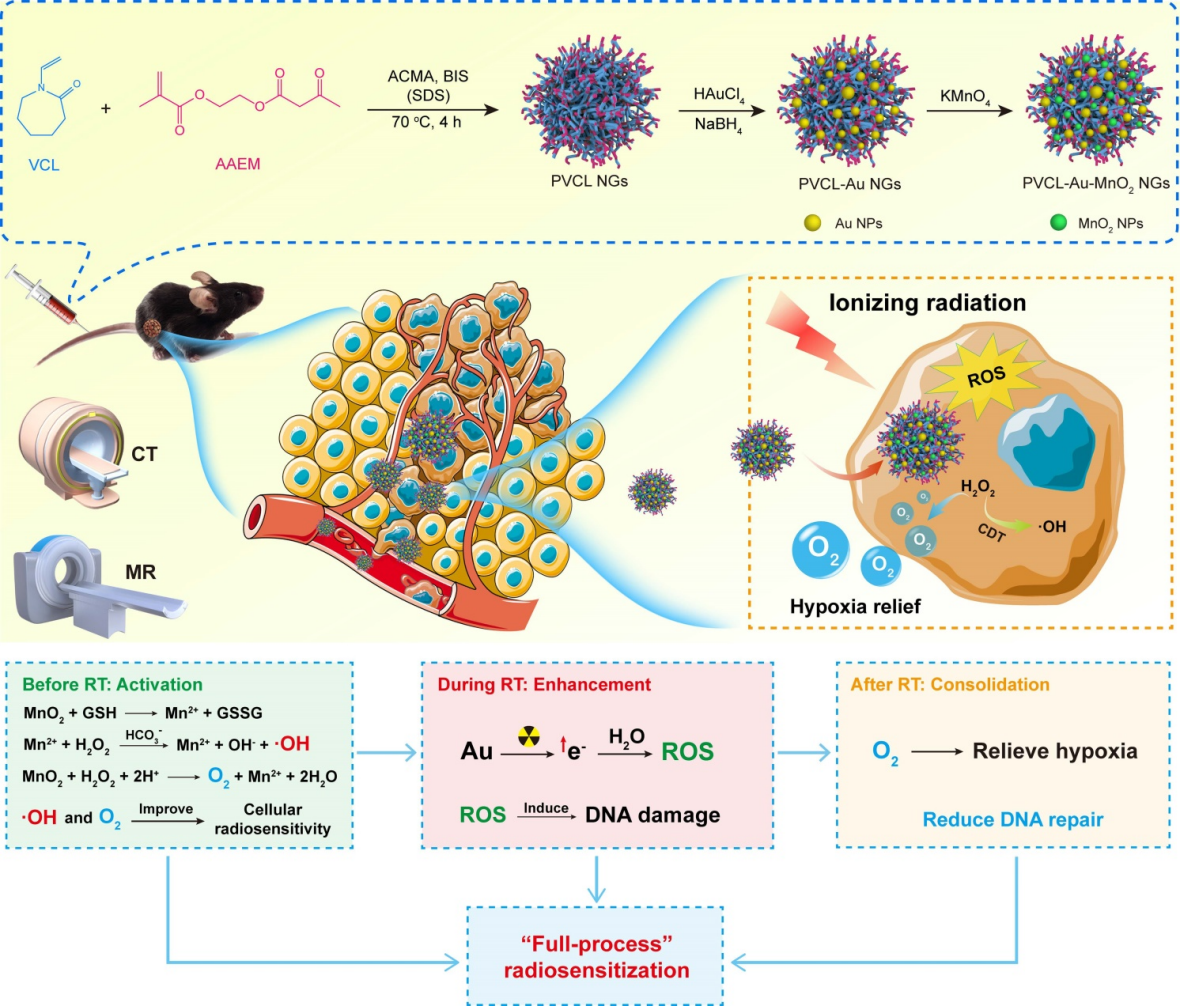
Synthesis of Au/MnO_2_-loaded PVCL NGs for enhanced RT *via* a full-process radiosensitization.

Herein, we report a facile design and synthesis of Au/MnO_2_-coloaded PVCL (PVCL-Au-MnO_2_) NGs for enhanced RT *via* a “full-process” radiosensitization (**Scheme 1**). PVCL NGs were firstly synthesized by a precipitation polymerization method, *in situ* immobilized with ultrasmall Au NPs through sodium borohydride (NaBH_4_) reduction chemistry to obtain PVCL-Au NGs, and then further *in-situ* loaded with MnO_2_ NPs through a mild oxidization with potassium permanganate (KMnO_4_). The created PVCL-Au-MnO_2_ NGs were systematically characterized, checked in terms of their cytotoxicity, cellular uptake, intracellular oxygen generation and ·OH production, and cell cycle arrest *in vitro*, and evaluated *in vitro* and *in vivo* to disclose their RT sensitization effects of cancer cells and a tumor model. Lastly, the dual-mode computed tomography (CT)/MR imaging potential and the *in-vivo* pharmacokinetics, biodistribution, and biosafety profile were also evaluated. To our knowledge, our current design owns distinct features: (I) the MnO_2_ component possesses ·OH production capacity through a Fenton-like reaction to regulate the cell cycle distribution toward the most radiosensitive G2/M phase before RT; (II) ultrasmall Au NPs in the NGs can concentrate the local radiation dose and amplify the X-ray induced DNA damage upon radiation treatment; (III) the continuous MnO_2_-catalyzed production of O_2_ relieves the tumor hypoxia, thus preventing the DNA-repairing process after RT treatment; and (IV) the hybrid NGs could be used as an efficient dual-mode CT/MR contrast agent for accurate tumor imaging.

## Results and Discussion

### Synthesis and characterization of PVCL-Au-MnO_2_ NGs

**Scheme 1** shows the synthesis steps of PVCL-Au-MnO_2_ NGs. First, PVCL NGs with 10 mol % acetoacetoxyethyl methacrylate (AAEM) as a comonomer were prepared *via* a precipitation polymerization method according to the literature 39. The adopted two-step AAEM addition is to ensure the equal distribution of β-diketone groups in the NGs, since AAEM has a higher polymerization speed than VCL, and would be mainly incorporated in the core of NGs otherwise 46. The resultant PVCL NGs served as a nanoreactor to first absorb Au(III) ions through interaction with the β-diketone groups, followed by fast reduction with NaBH_4_ to form Au NPs. Afterwards, *in situ* synthesis of MnO_2_ NPs was carried out by reducing KMnO_4_ utilizing β-diketone groups inside NGs as a mild reductant.

**Figure 1:**
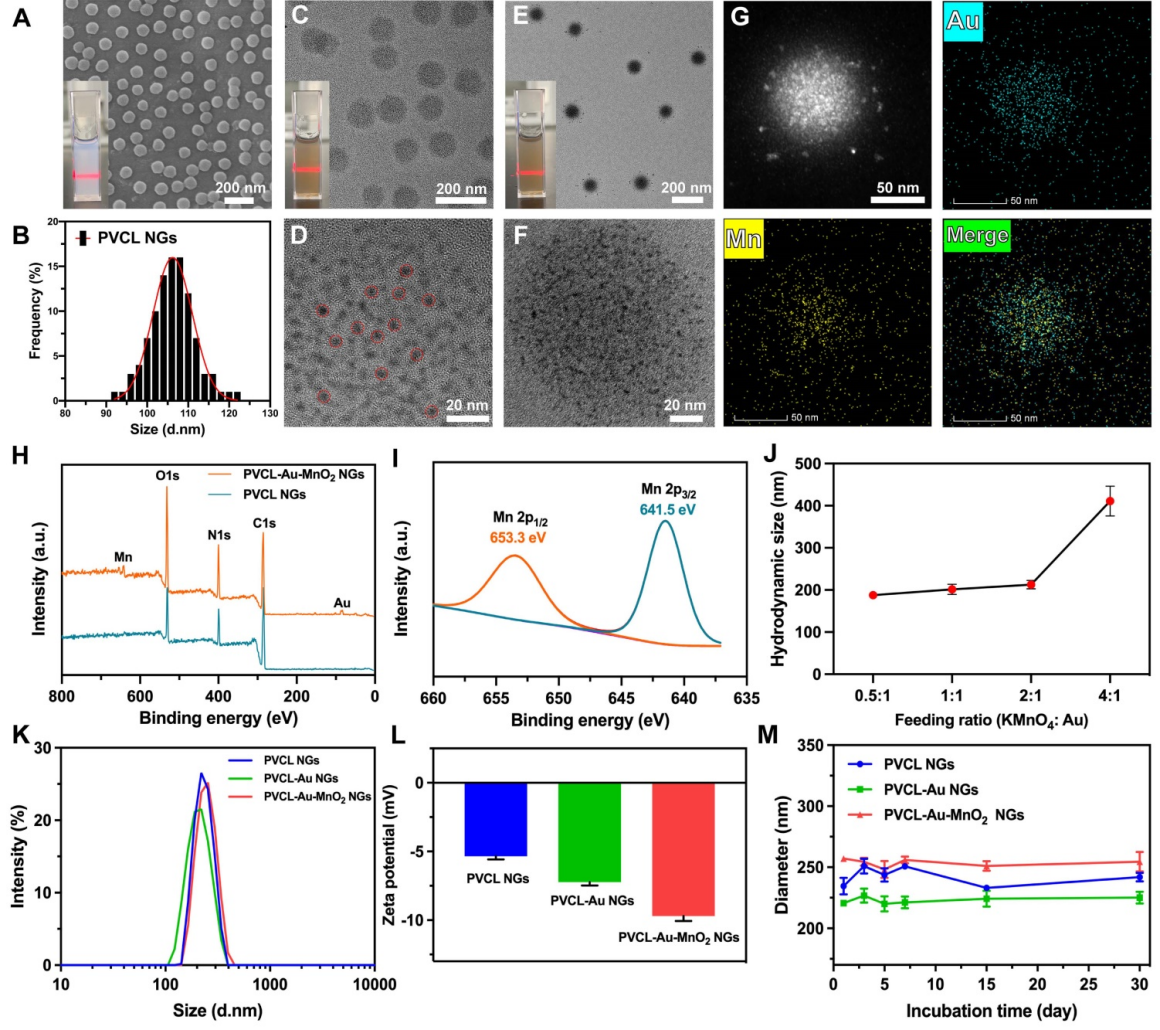
(A) SEM image of PVCL NGs (inset: digital photo of PVCL NGs dispersed in water). (B) Size distribution histogram of PVCL NGs. TEM images of (C) PVCL-Au NGs and (E) PVCL-Au-MnO_2_ NGs (insets: digital photos of the corresponding PVCL-Au and PVCL-Au-MnO_2_ NGs dispersed in water). High-resolution TEM images of (D) PVCL-Au and (F) PVCL-Au-MnO_2_ NGs. The red dotted circles in (D) show the single Au NPs in NGs. (G) Element mapping TEM images of PVCL-Au-MnO_2_ NGs. (H) XPS survey spectra of PVCL and PVCL-Au-MnO_2_ NGs. (I) High-resolution XPS spectrum of PVCL-Au-MnO_2_ NGs. (J) Hydrodynamic size of PVCL-Au-MnO_2_ NGs at different KMnO_4_/Au feeding mass ratios (n = 3). (K) Hydrodynamic size distribution and (L) ζ-potentials of PVCL, PVCL-Au and PVCL-Au-MnO_2_ NGs (n = 3). (M) Size changes of different NG formulations incubated with PBS containing 10% FBS for 30 days (n = 3).

As shown in **Figure 1A**, scanning electron microscope (SEM) image reveals the regular spherical shape and uniform size distribution of the obtained PVCL NGs. The size of PVCL NGs was measured to be 107.3 ± 2.4 nm (**Figure 1B**). Transmission electron microscope (TEM) was used to observe the PVCL-Au NGs after Au NP formation within the NGs (**Figure 1C**). The dried PVCL-Au NGs display a spherical shape with an average particle size of 116.6 ± 3.1 nm, and the successful incorporation of Au NPs (diameter = 4.2 ± 0.8 nm) inside the PVCL NGs (**Figure 1D**) can be confirmed by high-resolution TEM. As exhibited in **Figure 1E-F**, after further loading of MnO_2_ NPs (mass ratio of KMnO_4_: Au = 2: 1), the formed PVCL-Au-MnO_2_ NGs still have a uniform spherical shape but with a slightly larger diameter (121.5 ± 2.7 nm) than that before MnO_2_ NP loading (116.6 ± 3.1 nm). This suggests the success of the loading of MnO_2_ NPs. Unfortunately, due to the excellent hybridization and overlapping between Au and MnO_2_ NPs, it is very difficult to precisely determine the size of the MnO_2_ NPs within the NGs. As evidenced by SEM and TEM images, PVCL NGs before and after each step of NP loading display super water dispersibility, indicating their excellent cargo loading capacity.

To further confirm the loading of Au and MnO_2_ NPs within PVCL NGs, elemental mapping was performed (**Figure 1G**). Clearly, homogeneous distributions of Au and Mn contents in the PVCL-Au-MnO_2_ NGs can be observed, verifying the successful and uniform loading of Au and MnO_2_ NPs within the NGs. To investigate the detailed valence of Mn element, X-ray photoelectron spectroscopy (XPS) was undertaken (**Figure 1H-I**). The XPS survey spectrum shows the peaks of C, N and O elements for PVCL NGs (green curve), and peaks of Au, Mn, C, N, and O elements for the PVCL-Au-MnO_2_ NGs (orange curve), demonstrating the loading of Au and Mn elements inside the NGs (**Figure 1H**). The high-resolution XPS spectrum shows the Mn 2p peaks located at 653.3 and 641.5 eV, which can be derived from Mn 2p_1/2_ and Mn 2p_3/2_, respectively (**Figure 1I**), indicating the formation of MnO_2_, in agreement with the literature 27. This suggests that a mild reduction method is effective for the *in-situ* synthesis of MnO_2_ NPs within PVCL NGs.

We then investigated the MnO_2_ loading capacity of PVCL-Au NGs. Before that, we firstly quantified the Au content in the PVCL-Au NGs by inductively coupled plasma-optical emission spectroscopy (ICP-OES), and the loading content (LC) was measured to be 4.2%. With the increase of feeding mass ratio of KMnO_4_/Au, the size of PVCL-Au-MnO_2_ NGs slightly increases to 212.6 ± 7.6 nm at the mass ratio of 2: 1, followed by a sharp increase to 411.3 ± 35.6 nm at the ratio of 4: 1 (**Figure 1J**). The sharp increase of the NG size should be due to the severe aggregation of the NGs with the increased MnO_2_ loading that may exceed the loading capacity of the NGs. As shown in Table S1, the PVCL-Au NGs exhibits a loading efficiency (LE) of 89.1% and a loading content of 3.7% for MnO_2_ NPs at the KMnO_4_/Au mass ratio of 2: 1. As is reported by our previous work 47, NGs with a hydrodynamic size of ~200 nm display a better cellular uptake behavior and tumor penetration capacity than those with a size of 400 nm. In addition, to balance the optimized LE, LC, and the NG size for biomedical applications, we decided to select the NGs synthesized at the KMnO_4_/Au mass ratio of 2: 1 for further characterization and applications.

The hydrodynamic size and ζ-potential of the functional PVCL-Au-MnO_2_ NGs were monitored during each step of functionalization of PVCL NGs. As shown in **Figure 1K**, all the different NGs have hydrodynamic diameters of ~200-220 nm with a PDI of ~0.10, indicating the good water dispersity and uniformity of the as-prepared NGs with or without Au/MnO_2_ loading. The ζ-potential of PVCL NGs (-5.31 mV) is higher than that of PVCL-Au NGs (-7.25 mV), and changed to -9.73 mV after *in situ* generation of MnO_2_ NPs (**Figure 1L**). The slight change in ζ-potential demonstrates the successful incorporation of Au and MnO_2_ NPs in the PVCL NGs, in consistence with the literature 27. To ensure the long-term stability of the prepared hybrid NGs, which is crucial during systemic administration, we recorded their hydrodynamic size in phosphate buffered saline (PBS) containing 10% FBS for up to 30 days at room temperature. As expected, no significant size change of NGs was observed during the incubation time period (**Figure 1M**), indicating their excellent colloidal stability. Moreover, to evaluate the chemical stability of Au and MnO_2_ contents in the PVCL NGs, the leakage of Au and MnO_2_ contents under a simulated physiological environment (in PBS, pH 7.4, at 37 ^o^C) was monitored by ICP-OES. As revealed in **Figure S1**, after stored for 1, 3, 5 and 7 days, respectively, the leaked Au and Mn contents (% of original) are all less than 1%, indicating the superior chemical stability of Au and MnO_2_ in PVCL NGs.

### O_2_ generation capacity

**Figure 2:**
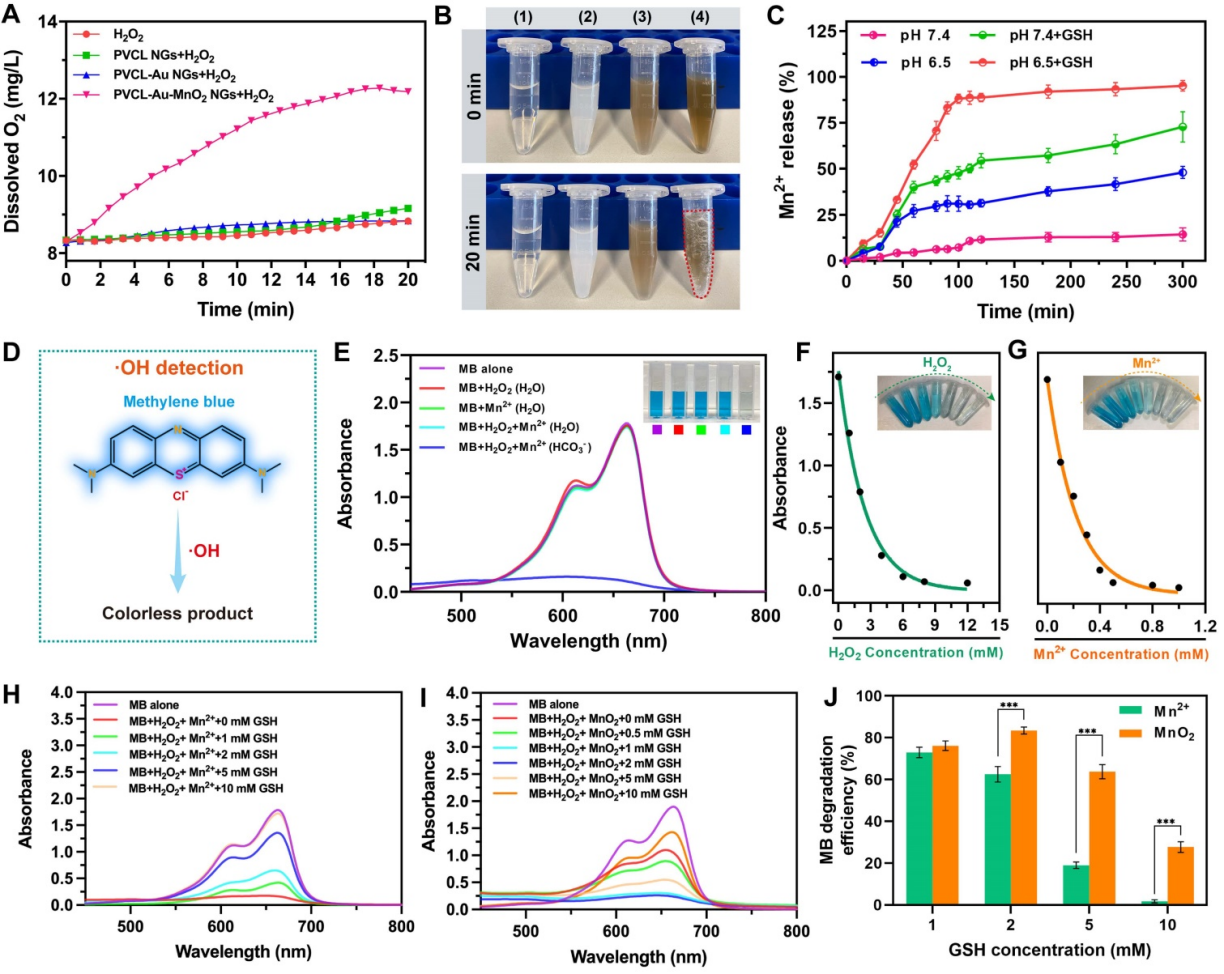
(A) O_2_ generation in water, PVCL, PVCL-Au and PVCL-Au-MnO_2_ NG dispersions (pH 6.5) in the presence of H_2_O_2_ (100 μM) as a function of incubation time. (B) Digital photos of (1) water, (2) PVCL NG, (3) PVCL-Au NG and (4) PVCL-Au-MnO_2_ NG dispersions (pH 6.5) incubated with H_2_O_2_ (500 μM) at 0 min (upper panel) and 20 min (bottom panel). (C) Mn^2+^ release from PVCL-Au-MnO_2_ NG dispersion containing 100 μM of H_2_O_2_ at different pHs (6.5 and 7.4) with or without GSH measured by a Spectroquant^®^ Mn^2+^ test kit (n = 3). (D) The reaction of blue-colored MB with ·OH to form colorless product (·OH detection). (E) UV-vis spectra and digital photo (inset) of MB treated in different solutions. Fitting of the MB absorbance at 665 nm as a function of (F) H_2_O_2_ and (G) Mn^2+^ concentration. (H) MB degradation by Mn^2+^-mediated Fenton-like reaction in the presence of GSH at different concentrations (0, 1, 2, 5 and 10 mM, respectively). (I) MB degradation by PVCL-Au-MnO_2_ NGs in the presence of GSH at different concentrations (0, 0.5, 1, 2, 5 and 10 mM, respectively). (J) MB degradation efficiency of Mn^2+^ and PVCL-Au-MnO_2_ NGs in the presence of GSH at different concentrations (1, 2, 5 and 10 mM, respectively, n = 3, and *** represents for *p* < 0.001).

It is known that under a mild acidic condition, MnO_2_ can catalyze the transformation of H_2_O_2_ to generate O_2_ 48. We therefore studied the O_2_ generation in the H_2_O_2_ solution (100 μM, pH 6.5) containing PVCL, PVCL-Au or PVCL-Au-MnO_2_ NGs using a multiparameter benchtop meter. As expected, the PVCL-Au-MnO_2_ NGs enable quick production of O_2_ due to the role played by the incorporated MnO_2_ as a catalyst, whereas no significant O_2_ production can be detected in the PVCL and PVCL-Au NG dispersions, as well as NG-free solution under the same incubation conditions for over 20 min (**Figure 2A**). Notably, to visualize the apparent O_2_ bubble formation (**Figure 2B**), we increased the H_2_O_2_ concentration up to 500 μM under other conditions fixed. The O_2_ bubble formation illustrates the superior capacity of PVCL-Au-MnO_2_ NGs to generate O_2_ by decomposing H_2_O_2_ under the slight acidic environment.

### Stimuli-responsive Mn^2+^ release

In comparison with normal tissues, TME possesses a lower pH, higher intracellular concentration of GSH and elevated level of H_2_O_2_ 49-51. We next evaluated the stimuli-responsive Mn^2+^ release behavior of PVCL-Au-MnO_2_ NGs. The PVCL-Au-MnO_2_ NGs were dispersed in phosphate buffer with H_2_O_2_ (100 μM) under pH 6.5 or 7.4 in the presence or absence of GSH (10 mM), and the released Mn^2+^ was determined by a Spectroquant^®^ Mn^2+^ test kit. As shown in **Figure 2C**, under pH 7.4 in the absence of GSH, negligible Mn^2+^ release (lower than 15%) was detected within 300 min. In contrast, under an acidic condition (pH 6.5), the Mn^2+^ release reaches ~50% in 300 min, indicating the promoting effect of H^+^ on the MnO_2_ degradation. Similarly, at the physiological pH and GSH (10 mM), the Mn^2+^ can be quickly released with ~70% in 300 min. As for the acidic condition (pH 6.5) plus 10 mM GSH, the release of Mn^2+^ almost reaches about 100% at 300 min, mainly attributed to the concurrent positive promotion of H^+^ and GSH to degrade MnO_2_ to fastly release Mn^2+^.

### ·OH generation by Mn^2+^-induced Fenton-like reaction

Recently, Mn^2+^-mediated Fenton-like reaction has been reported to be able to convert H_2_O_2_ into ·OH in the presence of HCO_3_^-^ 30, 32, 52. We then employed methylene blue (MB) for colorimetric analysis of the generation of ·OH. The detection mechanism 29 is mainly relied on the reaction of blue-colored MB with ·OH to yield a colorless product (**Figure 2D**). As shown in **Figure 2E**, after incubation with H_2_O_2_ (8 mM) and MnCl_2_ (0.5 mM) in NaHCO_3_/5% CO_2_ buffer for 30 min, the MB solution changes from blue to colorless and a sharp decrease in UV-vis absorption at 665 nm can be observed. In contrast, no obvious UV-vis absorption change was detected in the aqueous MB solution containing H_2_O_2_ (8 mM) and MnCl_2_ (0.5 mM). Moreover, H_2_O_2_ or Mn^2+^ alone in aqueous solution or NaHCO_3_/5% CO_2_ buffer has no effect on MB absorbance (**Figure 2E** and **Figure S2**), this means that the MB degradation must occur in the presence of H_2_O_2_ and MnCl_2_ in NaHCO_3_/5% CO_2_ buffer.

Additionally, the Mn^2+^-mediated Fenton-like reaction to generate ·OH follows a H_2_O_2_- and Mn^2+^- concentration dependent manner (**Figure 2F**-**G**). The MB degradation can be tremendously inhibited by GSH with a scavenging effect on ·OH generated by Mn^2+^-mediated Fenton-like reaction (**Figure 2H**). Interestingly, the MB degradation efficiency in PVCL-Au-MnO_2_ NG dispersion gradually increases with the concentration of GSH from 0 to 2 mM, but decreases with excessive increase of GSH (5 mM or above), owing to its ·OH scavenging effect at a higher concentration (**Figure 2I**). At an excessive GSH concentration (10 mM), PVCL-Au-MnO_2_ NGs still exhibit an MB degradation efficiency of 27.67%, which is much higher than that of Mn^2+^ (1.72%, **Figure 2J**). This should be due to the GSH depletion effect of MnO_2_ 26, partially decreasing the GSH scavenging effect of ·OH. These results taken together indicate that the PVCL-Au-MnO_2_ NGs possess a better performance in ·OH generation and GSH depletion than free Mn^2+^.

Electron spin resonance was also used to directly identify the reactive oxygen species (ROS) generation ability of PVCL-Au-MnO_2_ NGs to induce ·OH production. 5,5-Dimethyl-1-pyrroline N-oxide was used as a trapping agent. As shown in **Figure S3**, the four-line spectra with relative intensity ratio of 1: 2: 2: 1 can be observed, and the signal intensity increases gradually with the incubation time. These results demonstrate that the developed PVCL-Au-MnO_2_ NGs are able to generate ·OH in a time-dependent manner.

### *In vitro* MR imaging performance of PVCL-Au-MnO_2_ NGs

Owing to the TME-responsive release of Mn^2+^ from the PVCL-Au-MnO_2_ NGs, we then evaluated the potential of PVCL-Au-MnO_2_ NGs as a TME-activated T_1_-weighted MR contrast agent. As revealed in **Figure S4**, the longitudinal relaxivity (r_1_) value of PVCL-Au-MnO_2_ NG solution at pH = 7.4 is only 0.06 mM^-1^s^-1^, much lower than that in an acidic solution (pH = 6.5, r_1_ = 3.37 mM^-1^s^-1^), due to the pH-responsive release of Mn^2+^ with improved T_1_-weighted MR contrast enhancement. Additionally, the PVCL-Au-MnO_2_ NGs under pH 7.4 plus GSH (10 mM) exhibit a higher r_1_ of 6.69 mM^-1^s^-1^, suggesting that the presence of GSH could more significantly promote Mn^2+^ release to improve their r_1_ value. Furthermore, in the simulated TME condition (pH 6.5, H_2_O_2_ = 100 μM, and GSH = 10 mM), the r_1_ value of the NGs is remarkably enhanced from the initial value of 0.06 mM^-1^s^-1^ (pH 7.4) to 14.04 mM^-1^s^-1^, due to the thorough decomposition of MnO_2_ content in PVCL-Au-MnO_2_ NGs to fastly release Mn^2+^. The acidity-induced and H_2_O_2_- and GSH-accelerated MnO_2_ degradation could be explained by the following reactions 53:



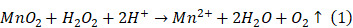





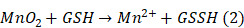



These results indicate the great potential of PVCL-Au-MnO_2_ NGs to be used as a TME-triggered contrast agent for T_1_-weighted MR imaging of tumors.

### *In vitro* cytotoxicity and cellular uptake assays

**Figure 3:**
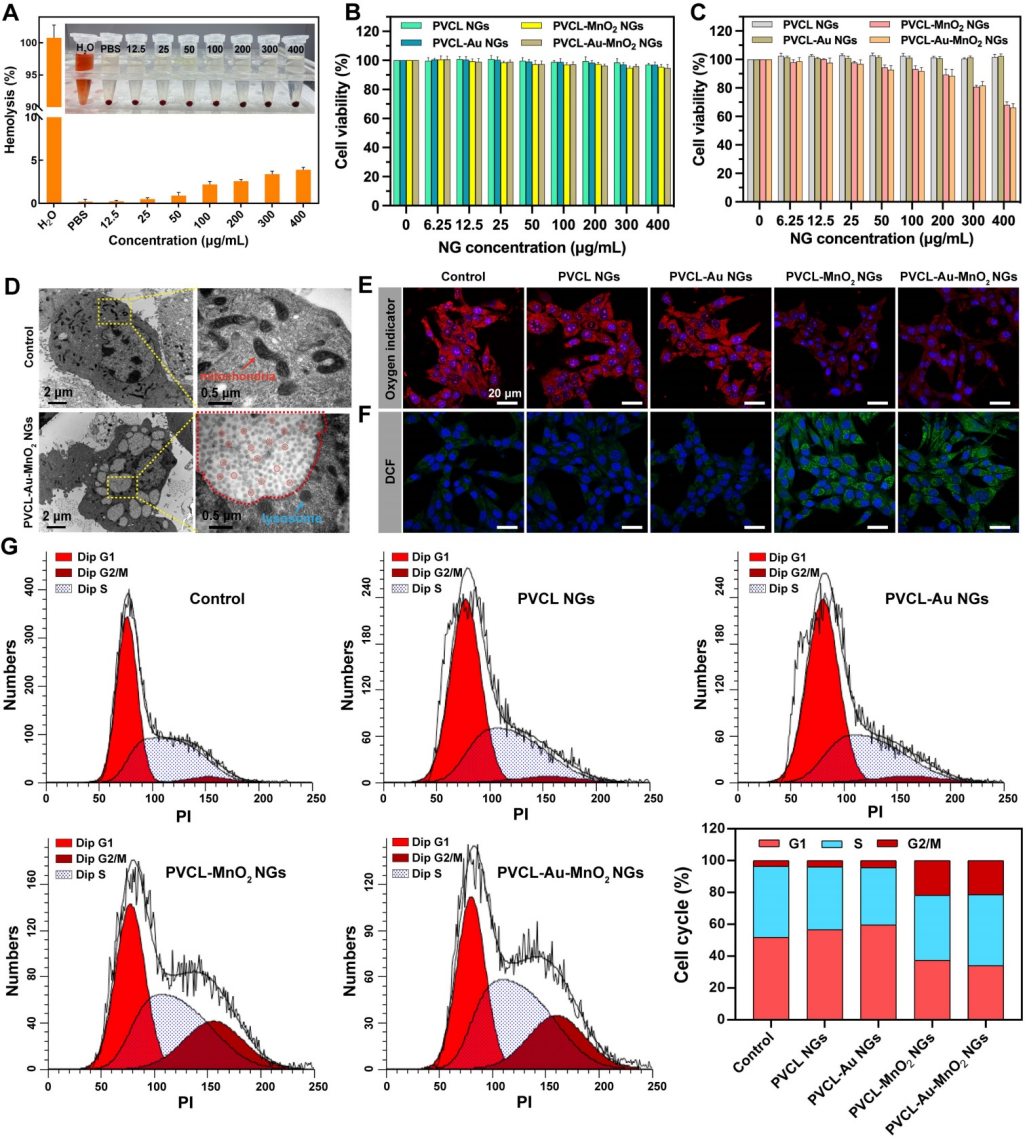
(A) Hemolysis percentage of red blood cells (RBCs) treated with PVCL-Au-MnO_2_ NGs at various concentrations for 2 h (n = 3). Inset shows the photograph of RBCs treated with the hybrid NGs at different concentrations, followed by centrifugation. Water and PBS were used as positive and negative controls, respectively. Viability of (B) L929 and (C) Pan02 cells after 24 h of incubation with PVCL, PVCL-Au, PVCL-MnO_2_ or PVCL-Au-MnO_2_ NGs (n = 4). (D) Bio-TEM images of Pan02 cells incubated with PVCL-Au-MnO_2_ NGs for 12 h (red box indicate the internalized NGs). CLSM images of intracellular (E) O_2_ and (F) ROS generation after 12 h of incubation with different NG formulations at an NG concentration of 200 μg mL^-1^. (G) Flow cytometric analysis of cell cycle distribution of Pan02 cells in different treatment groups.

Before biomedical applications, the blood compatibility of PVCL-Au-MnO_2_ NGs was evaluated. No obvious hemolytic effect (less than 5%) of PVCL-Au-MnO_2_ NGs can be observed even when the NG concentration is up to 400 μg mL^-1^ (**Figure 3A**), indicating their good hemocompatibility. Then, the cytotoxicity of PVCL, PVCL-Au, PVCL-MnO_2_ and PVCL-Au-MnO_2_ NGs were evaluated* via* CCK-8 assay of the viability of L929 and Pan02 cells. As shown in **Figure 3B**, L929 cells incubated with various NGs at a concentration as high as 400 μg mL^-1^ show negligible viability changes when compared to the PBS control, indicating the desirable cytocompatibility of these NGs. As for Pan02 cells, they remain alive after being incubated with Mn-free PVCL and PVCL-Au NGs at a concentration as high as 400 μg mL^-1^ (**Figure 3C**), indicating that these two kinds of NGs have no killing effect on cancer cells. Notably, the cells treated with the Mn-containing PVCL-MnO_2_ or PVCL-Au-MnO_2_ NGs exhibit a concentration-dependent viability decrease, with an inhibition rate of ~34% at 400 μg mL^-1^. This inhibition effect might be due to the fact that MnO_2_ NPs loaded in the NGs could efficiently convert H_2_O_2_ into highly toxic ·OH in cancer cell microenvironment, thus inducing the effective cell apoptosis. In order to better investigate the mechanism of the “full-process” radiosensitization, the NG concentration of 200 μg mL^-1^ that can achieve the cell viability over 85% was selected for subsequent experiments.

The cellular uptake behaviors of PVCL-Au-MnO_2_ NGs were then investigated. The bio-TEM images reveal that a large amount of PVCL-Au-MnO_2_ NGs are located in the endocytic vesicles, indicating the effective internalization of PVCL-Au-MnO_2_ NGs possibly through phagocytosis and diffusion pathways (**Figure 3D**). Furthermore, ICP-OES was performed to quantify the cellular uptake of PVCL-Au-MnO_2_ NGs. As shown in **Figure S5**, the Au and Mn amounts taken up by Pan02 cells gradually increase with the extension of incubation time, in agreement with the literature 43. The efficient cellular uptake and internalization of the PVCL-Au-MnO_2_ NGs should be beneficial for sensitized RT of cancer cells.

### Intracellular O_2_ and ·OH generation

We next evaluated the intracellular O_2_ production catalyzed by PVCL-Au-MnO_2_ NGs. [Ru(dpp)_3_]Cl_2_ whose fluorescence could be quenched by O_2_ was chosen as an O_2_ indicator 54. As shown in **Figure 3E and Figure S6**, the red fluorescence of [Ru(dpp)_3_]Cl_2_ in Pan02 cells incubated with the PVCL-MnO_2_ or PVCL-Au-MnO_2_ NGs is significantly quenched, and is much weaker than that in other groups treated with PBS, PVCL NGs or PVCL-Au NGs. This can be ascribed to the CAT-like activity of PVCL-MnO_2_ and PVCL-Au-MnO_2_ NGs, which could efficiently decompose the H_2_O_2_ in cancer cells to produce O_2_. Moreover, the relief of cellular hypoxia by PVCL-Au-MnO_2_ NG-induced O_2_ in Pan02 cells was further supported by the downregulation of hypoxia-inducible factor (HIF-1α) level after incubation with PVCL-Au-MnO_2_ NGs (**Figure S7**).

Furthermore, the intracellular ·OH generation was determined using a chemical probe 2',7'-dichlorofluorescin diacetate (DCFH-DA), which could be oxidized by ROS to form 2,7-dichlorofluorescein (DCF) with green fluorescence emission (**Figure 3F and Figure S8**). The groups of control, PVCL NGs, and PVCL-Au NGs show negligible green fluorescence. In contrast, Pan02 cells treated with PVCL-MnO_2_ and PVCL-Au-MnO_2_ NGs show prominent green fluorescence emission, which could be attributed to the generation of cellular ·OH by the Fenton-like reaction offered by Mn^2+^ released from the NGs through decomposition of MnO_2_.

### *In vitro* cell cycle arrest

It has been reported that the ROS generated by some exogenous drugs could arrest the cell cycle at G2/M phase, during which the cells are highly sensitive to radiation therapy 55-57. Therefore, we investigated whether the PVCL-Au-MnO_2_ NGs were able to regulate the cell cycle arrest at the G2/M phase through ·OH generation in cancer cells. The cell cycle distribution of Pan02 cells treated with different NGs was analyzed by flow cytometry (**Figure 3G**). As opposed to the groups of PBS, PVCL and PVCL-Au NGs, in the groups of PVCL-MnO_2_ and PVCL-Au-MnO_2_ NGs, the percentages of the cell population at G2/M phases remarkably increase by ~17%. These results indicate that the MnO_2_-loaded PVCL NGs possess an ability to regulate the cell cycle distribution toward the highly radiosensitive G2/M phase likely through the Mn^2+^-induced CDT.

### *In vitro* sensitized RT of cancer cells using PVCL-Au-MnO_2_ NGs

**Figure 4:**
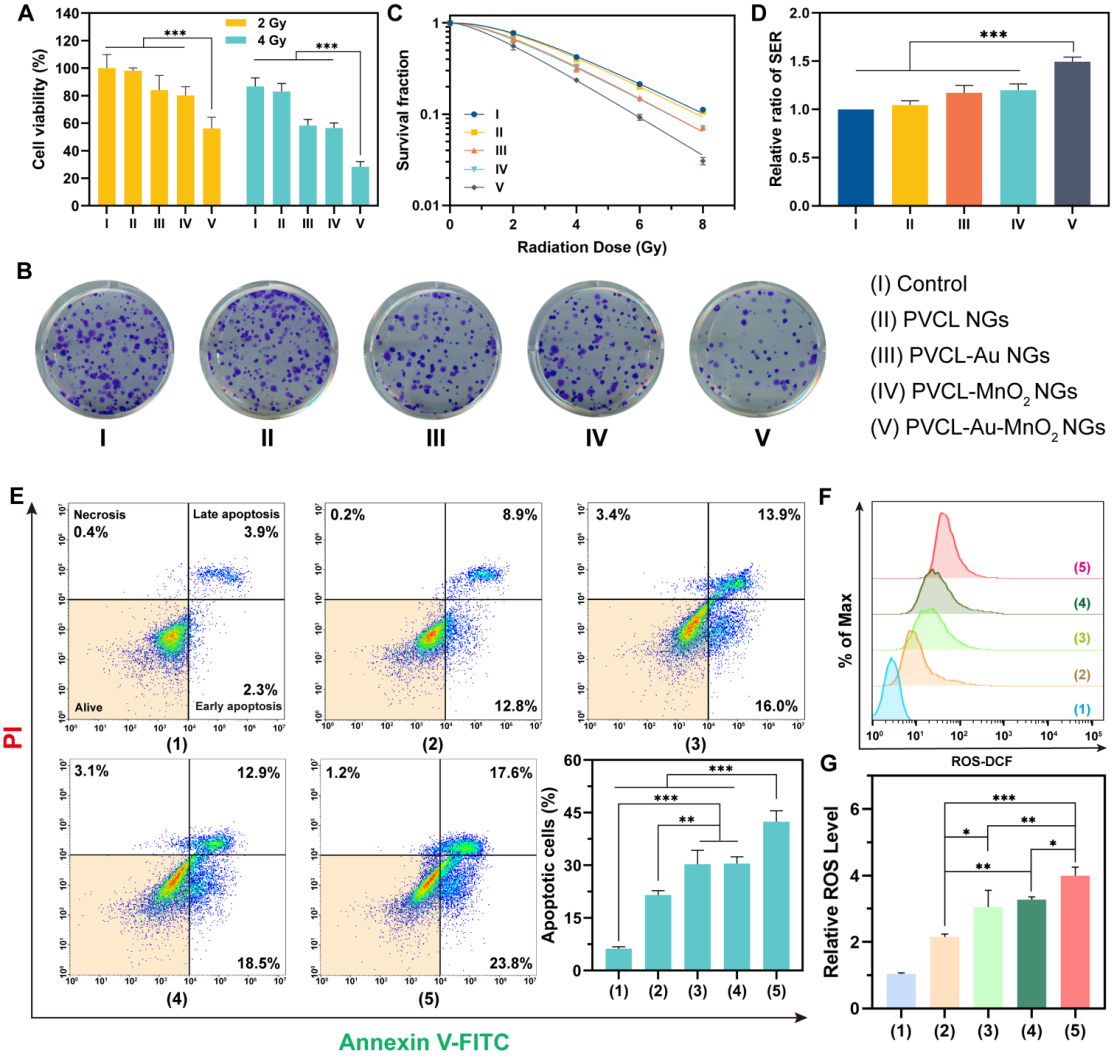
(A) Viability of Pan02 cells incubated with different NG formulations under X-ray irradiation (2 or 4 Gy). (B) The corresponding colony formation assay of Pan02 cells treated with different NGs plus 4 Gy of X-ray. (C) Survival fraction of NG-treated Pan02 cells under different doses of X-ray (0, 2, 4, 6 or 8 Gy). (D) SER of each group calculated by the multitarget single-hit model. (E) Flow cytometric assay of Pan02 cells and corresponding percentage of apoptotic cells under different treatments. (F, G) Flow cytometric analysis of ROS level in Pan02 cells after different treatments. The treatments are as follows: (1) PBS, (2) 4 Gy X-ray alone, (3) PVCL-Au NGs plus 4 Gy X-ray, (4) PVCL-MnO_2_ NGs plus 4 Gy X-ray, and (5) PVCL-Au-MnO_2_ NGs plus 4 Gy X-ray. In (A, D, E and G), n = 3 for each sample (*** for *p* < 0.001, ** for *p* < 0.01, and * for *p* < 0.05, respectively).

Inspired by the excellent properties of PVCL-Au-MnO_2_ NGs, their radiosensitization-enhanced therapeutic effect was then evaluated *in vitro*. Initially, the viability of Pan02 cells treated with the NGs upon X-ray irradiation with different doses (2 and 4 Gy) was determined. As shown in **Figure 4A**, the increase of X-ray radiation dose seems to decrease the cell viability in different degrees depending on the used NG types. The cell viability in the PBS control group (Group I) plus 2 Gy of X-ray was set to 100% as a base for comparisons. The cell viability in the PVCL NGs group (Group II) plus X-ray decreases from 98.2% (2 Gy) to 83.1% (4 Gy), while that in the PVCL-Au NGs (Group III) and PVCL-MnO_2_ NGs (Group IV) groups plus X-ray decreases from 84.1% (2 Gy) to 58.2 % (4 Gy), and 80.1% (2 Gy) to 56.5% (4 Gy), respectively. These results suggest that the PVCL-Au and PVCL-MnO_2_ NGs are able to increase the cellular radiosensitivity owing to the high-Z element of Au-induced dose enhancement effect, the hypoxia relief and CDT (·OH generation) effect from MnO_2_, respectively. The latter case appears to be more significant than the former one based on the high-Z element of Au. Furthermore, the cell viability in the PVCL-Au-MnO_2_ NGs group (Group V) plus X-ray displays a significant decrease with an inhibition rate as high as 71.7% on Pan02 cells at 4 Gy, likely attributing to the combination radiosensitization effect derived from both Au and MnO_2_ within the NGs.

We next investigated the effects of the hybrid NGs on the proliferation of Pan02 cells using clonogenic survival assays. As presented in **Figure 4B-C**, the colony formation rates of Pan02 cells in Groups I and II after treatment with 4 Gy radiation are 42.4% and 40.1%, respectively, while the cell survival fractions of Groups III and IV decrease to 32.2% and 30.7%, respectively. Moreover, the cells in Group V after treatment with X-ray display a high colony inhibition to have an extremely low cell survival rate of 18.6%, indicating the superior radiosensitization and killing effect of PVCL-Au-MnO_2_ NGs. The clonogenic survival assays also reveal the dose-dependent radiosensitization effects of the hybrid NGs (**Figure 4C**). Next, we calculated the sensitization enhancement ratios (SER) to evaluate the radiosensitization efficiency using the multitarget single-hit model. Taking the control group (Group I) plus 4 Gy radiation as the standard (1.00), the SER for cells irradiated in the presence of PVCL-Au-MnO_2_ NGs (Group V) was calculated to be ~1.49, which is obviously much higher than those of other groups (*p* < 0.001, **Figure 4D**).

Furthermore, we conducted the annexin V-FITC/PI double staining to study the cell apoptosis induced by RT (**Figure 4E**). The PBS control group (Group 1) displays almost no cell apoptosis, whereas a significant proportion of apoptotic cells (21.5%) can be detected in the group treated with X-ray alone (Group 2), illustrating the obvious apoptotic effect of RT on cancer cells. Importantly, when a combination of X-ray with PVCL-Au NGs (Group 3) and PVCL-MnO_2_ NGs (Group 4) was used, the apoptotic rate drastically increases to 30.3% and 31.5%, respectively, revealing the respective radiosensitization effects of PVCL-Au and PVCL-MnO_2_ NGs. Moreover, the group of PVCL-Au-MnO_2_ NGs plus 4 Gy X-ray (Group 5) has the highest apoptotic rate of ~42.4%. These results show that the PVCL-Au-MnO_2_ NGs could serve as a promising radiosensitizer for enhanced RT of cancer cells.

To further investigate the intracellular mechanisms of the hybrid NG-mediated enhanced RT, we evaluated the intracellular ROS level in each treatment group after X-ray irradiation using the ROS detection probe DCFH-DA, since the elevated ROS after ionization irradiation is considered to play an important role in promoting cell apoptosis during RT 15, 58. It is accepted that the generated ROS tends to break the vital chemical bonds of DNA, thus inducing programmed cell death 59, 60. As shown in **Figure 4F-G**, the intracellular ROS level increases by 2.1-fold for cells in Group 2 compared with the PBS control group (Group 1), confirming the ROS generation by ionization irradiation. Interestingly, the cellular ROS level in Group 3 is 3.1-fold higher than Group 1, which might be ascribed to the electronically active Au NPs in the NGs 58, 61. Similarly, a 3.3-fold increase in ROS level can be observed for cells in Group 4 owing to the combined effect of MnO_2_-mediated CDT and ionization irradiation. As expected, the PVCL-Au-MnO_2_ NGs plus X-ray (Group 5) induce the highest ROS generation.

**Figure 5:**
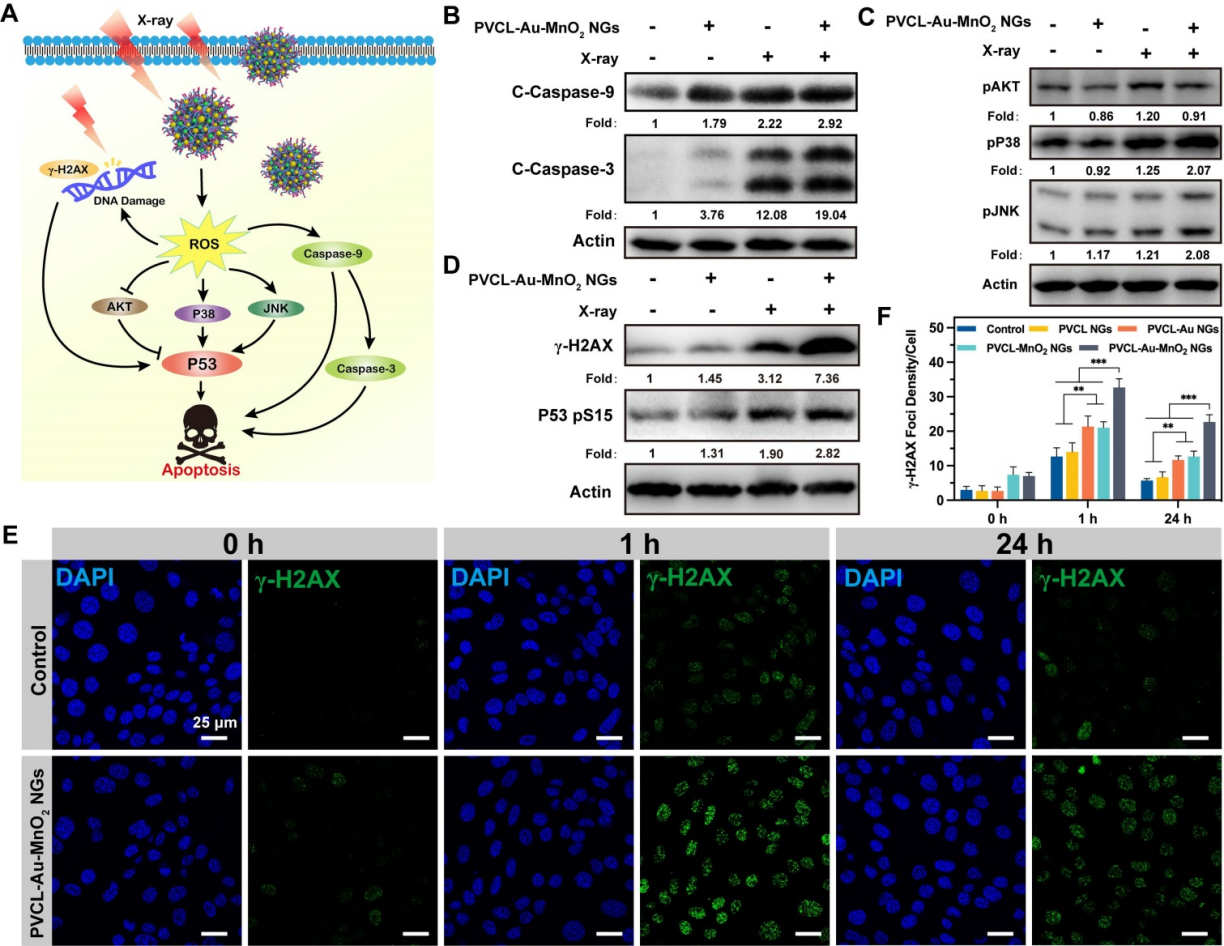
(A) Proposed molecular mechanism for the enhanced cell death triggered by PVCL-Au-MnO_2_ NGs and X-ray irradiation. Western blot analysis of the expression or phosphorylation of the (B) cleaved caspase-9/-3, (C) AKT and MAPKs signaling, and (D) γ-H2AX as well as p53 in Pan02 cells after incubation with PVCL-Au-MnO_2_ NGs (200 μg mL^-1^) with (+) and without (-) X-ray (4 Gy). (E) Change of γ-H2AX foci (green) in cell nuclei (blue) of Pan02 cells treated with PVCL-Au-MnO_2_ NGs plus X-ray (4 Gy) or by X-ray alone (control). (F) The corresponding γ-H2AX foci density in Pan02 cells at different time points post X-ray irradiation (n = 5, *** for *p* < 0.001 and ** for *p* < 0.01, respectively).

We then studied deeply the underlying molecular mechanism of PVCL-Au-MnO_2_ NG-induced radiosensitization efficacy. The proposed signaling pathways induced by PVCL-Au-MnO_2_ NGs plus X-ray are presented in **Figure 5A**. Since the caspase family members play a central role in programmed cell death or apoptosis 62-64, we first evaluated the expression levels of the typical caspases by western blotting. As displayed in **Figure 5B**, the obviously elevated expression levels of cleaved caspase-3 and cleaved caspase-9 in Pan02 cells after treatment with PVCL-Au-MnO_2_ NGs and X-ray suggest that the enhanced apoptotic cell damage is achieved by the activation of caspase-induced apoptosis pathway. Meanwhile, the protein kinase B (AKT) and mitogen-activated protein kinases (MAPKs), which are the downstream signaling pathways of ROS overproduction that is essential in cell proliferation, migration and apoptosis were also evaluated. As shown in **Figure 5C**, the PVCL-Au-MnO_2_ NGs plus X-ray slightly reduce the AKT phosphorylation level and upregulate the expression levels of phosphorylated p38 and Jun N-terminal kinase (JNK). Moreover, the expression of p53 and DNA damage-related proteins such as phospho-histone H2AX (γ-H2AX) in Pan02 cells were determined to gain further insight into the mechanism of apoptosis induced by PVCL-Au-MnO_2_ NGs and X-ray. The western blot results in **Figure 5D** reveal that the combination of PVCL-Au-MnO_2_ NGs and X-ray significantly increases the DNA damage by elevating the expression of γ-H2AX and phosphorylated P53. The foregoing results evidently demonstrate that the death of Pan02 cells after treatment of PVCL-Au-MnO_2_ NGs plus X-ray is caused by the synergistic effects arising from the activation of caspases-, p53-, and DNA damage-mediated apoptosis pathways.

To further study the DNA damage enhancement and DNA repair prevention effects after treatment with PVCL-Au-MnO_2_ NGs plus X-ray, the DNA double-strand breaks were detected at different time points (0, 1 or 24 h) post-treatments by γ-H2AX analysis. As shown in **Figure 5E-F** and **Figure S9**, upon 4 Gy X-ray irradiation, the DNA damage level in the PVCL NGs group is comparable to that in the PBS control group at 1 h post-treatment, revealing that the PVCL NGs have no effect on DNA damage. Meanwhile, enhanced DNA damages in Pan02 cells can be observed in PVCL-Au NGs plus X-ray and PVCL-MnO_2_ NGs plus X-ray groups, suggesting that the Au or MnO_2_ NPs could boost the cellular radiosensitization, facilitating enhanced DNA damages. Notably, the Pan02 cells treated with PVCL-Au-MnO_2_ NGs plus X-ray show the strongest γ-H2AX fluorescence, indicating the highest DNA damage among all groups. Moreover, at 24 h post-treatment, the γ-H2AX foci density in each group shows different degrees of decrease, indicating the DNA repair in Pan02 cells. The DNA repair rates of Pan02 cells treated with X-ray alone (control group) or PVCL NGs plus X-ray irradiation were calculated to be 55.9% and 52.1%, respectively. When the treatment of X-ray was combined with PVCL-Au, PVCL-MnO_2_, and PVCL-Au-MnO_2_ NGs, the DNA repair rates decrease to 45.1%, 39.5%, and 30.6%, respectively. Taken together, the above results suggest that the developed PVCL-Au-MnO_2_ NGs are able to enhance DNA damage of cancer cells during RT by improving the cellular radiosensitivity and significantly preventing the rapid DNA repair after RT, resulting in efficient therapeutic outcome.

### Pharmacokinetics and *in vivo* biodistribution studies

Before biomedical applications, it is crucial to investigate the pharmacokinetics of PVCL-Au-MnO_2_ NGs. As shown in **Figure S10**, the half-decay time (t_1/2_) of PVCL-Au-MnO_2_ NGs was calculated to be 1.38 h, which is relatively long and would benefit the enhanced permeability and retention (EPR)-based passive accumulation of NGs in tumor sites. Meanwhile, the biodistribution of Au elements in major organs and tumors at different time points post-injection was analyzed to track the metabolization pathway of PVCL-Au-MnO_2_ NGs *in vivo* (**Figure S11**). A large amount of Au elements is observed in the liver and spleen at 6 or 12 h post intravenous (i.v.) administration, which then gradually decreases with the time post-injection, revealing the uptake and elimination of NGs by reticuloendothelial system organs. Additionally, the PVCL-Au-MnO_2_ NGs could be efficiently accumulated in the tumor region with a relatively high amount of 12.37% at 24 h post-injection by the passive EPR effect, suggesting that the ideal radiation time point can be selected at around 24 h post-administration.

### Dual-mode CT/MR imaging of tumors

**Figure 6:**
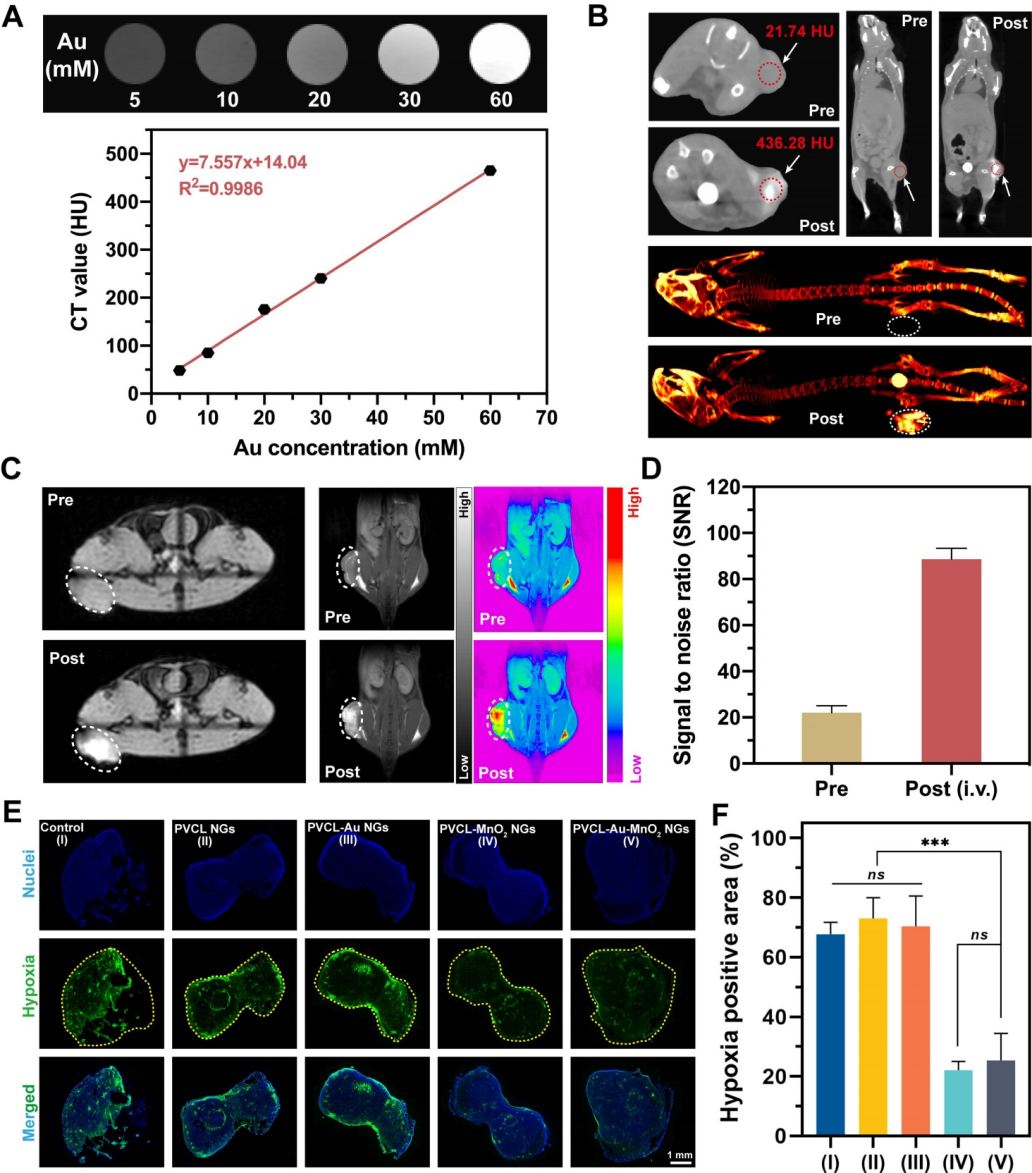
(A) *In vitro* CT images and CT values of PVCL-Au-MnO_2_ NG aqueous solutions with different Au concentrations. (B) *In vivo* CT images of Pan02 tumor-bearing mice before and at 24 h post i.v. injection with PVCL-Au-MnO_2_ NGs ([Au] = 10 mM, in 100 μL of PBS for each mouse). (C) MR imaging of tumor-bearing mice before injection and at 24 h post i.v. administration ([Mn] = 10 mM, in 100 μL of PBS for each mouse) and (D) corresponding MR SNR values of the tumors (n = 3). The tumor sites were circled by white or red dashed line for Panel (B) or (C). (E) Hypoxia-positive immunofluorescence images of tumor slices and (F) quantitative analysis of hypoxia relief in tumor sites (n = 3). The yellow dashed lines indicate the tumor boundaries. *** represents *p* < 0.001, and *ns* indicates *p* > 0.05, respectively.

Because of the high atomic number and strong X-ray attenuation coefficient of Au element, we performed CT phantom studies of the PVC L-Au-MnO_2_ NGs to check their potential to be used as a CT contrast agent. As shown in **Figure 6A**, the Hounsfield unit (HU) value increases linearly with the Au concentration at a rate of 7.557 HU mM^-1^, showing the great potential to use the hybrid NGs for CT imaging. Then, the CT imaging potential of PVCL-Au-MnO_2_ NGs was assessed in a xenografted pancreatic tumor model *in vivo* after i.v. injection. As is evident from **Figure 6B**, a significant CT signal enhancement at the tumor site can be achieved at 24 h post-injection. The tumor CT value obviously increases from 21.7 ± 1.6 HU to 436.3 ± 3.8 HU, indicating that the PVCL-Au-MnO_2_ NGs could be effectively accumulated in the tumor region *via* EPR effect for efficient tumor CT imaging. It is interesting to note that although the tumor tissue shows the apparent CT signal, the liver and spleen organs do not seem to have similar CT signal intensity at the same time point of 24 h post-injection although the biodistribution data show the close Au uptake between liver/spleen and tumor (**Figure S11**). This is likely due to the fact that there may be some artifact elements absorbing the X-ray to false negatively impacting the X-ray attenuation of deep liver and spleen organs, in agreement with the literature 54.

As encouraged by the TME-responsive MR imaging performance, the potency of PVCL-Au-MnO_2_ NGs as an MR contrast agent was next investigated *in vivo* (**Figure 6C-D**). At 24 h post i.v. administration, the MR signal intensity at the tumor site is much higher than that before injection. Quantitative MR signal-to-noise ratio (SNR) analysis exhibits a 4.1-fold increase when compared to that before injection. This suggests the feasibility to use the developed PVCL-Au-MnO_2_ NGs as an ideal contrast agent for activatable MR imaging of tumors. It should be noted that the Mn^2+^-containing NGs could stay at the tumor site for a certain period of time for efficient MR imaging of tumors regardless of the quick diffusion of free Mn^2+^ ions. This is because the conversion of MnO_2_ NPs to Mn^2+^ in the heterogeneous TME is a dynamic process, and the Mn^2+^ ions can be sustainably created for a certain period of time for MR imaging. It is also interesting to note that we chose 24 h post-injection as an ideal time point for tumor CT/MR dual mode imaging just because the accumulation of PVCL-Au-MnO_2_ NGs in the tumor site reached a peak value at 24 h post i.v. injection (**Figure S11**).

### Hypoxia relief *in vivo*

Stimulated by the hypoxia relief capacity of PVCL-Au-MnO_2_ NGs in cellular levels and their superior tumor accumulation ability evidenced by dual-mode CT/MR imaging *in vivo*, we next conducted animal experiments to explore whether the hybrid NGs could relieve the tumor hypoxia *in vivo*. Notably, we find that the tumors treated with MnO_2_-containing NGs (PVCL-MnO_2_ and PVCL-Au-MnO_2_ NGs) show much weaker hypoxia-positive fluorescence intensities than those treated with PBS, PVCL NGs, and PVCL-Au NGs (**Figure 6E**). This is mainly ascribed to the elevated O_2_ generation by the incorporated MnO_2_ within the NGs under the TME. From the quantitative hypoxia-positive fluorescence intensity analysis (**Figure 6F**), it can be clearly seen that the percentage of hypoxia-positive tumor area dramatically decreases from 67.6% for PBS control group to 22.4% and 25.3% for the PVCL-MnO_2_ and PVCL-Au-MnO_2_ NGs groups, respectively (*p* < 0.001). The hypoxia-positive tumor area in the PVCL and PVCL-Au NGs groups shows no obvious difference compared with the PBS control group (*p* > 0.05). These results confirm that the PVCL-Au-MnO_2_ NGs could be used as a promising agent to relieve tumor hypoxia for sensitized tumor RT.

### *In vivo* “full-process” sensitized RT of Pan02 tumors

**Figure 7:**
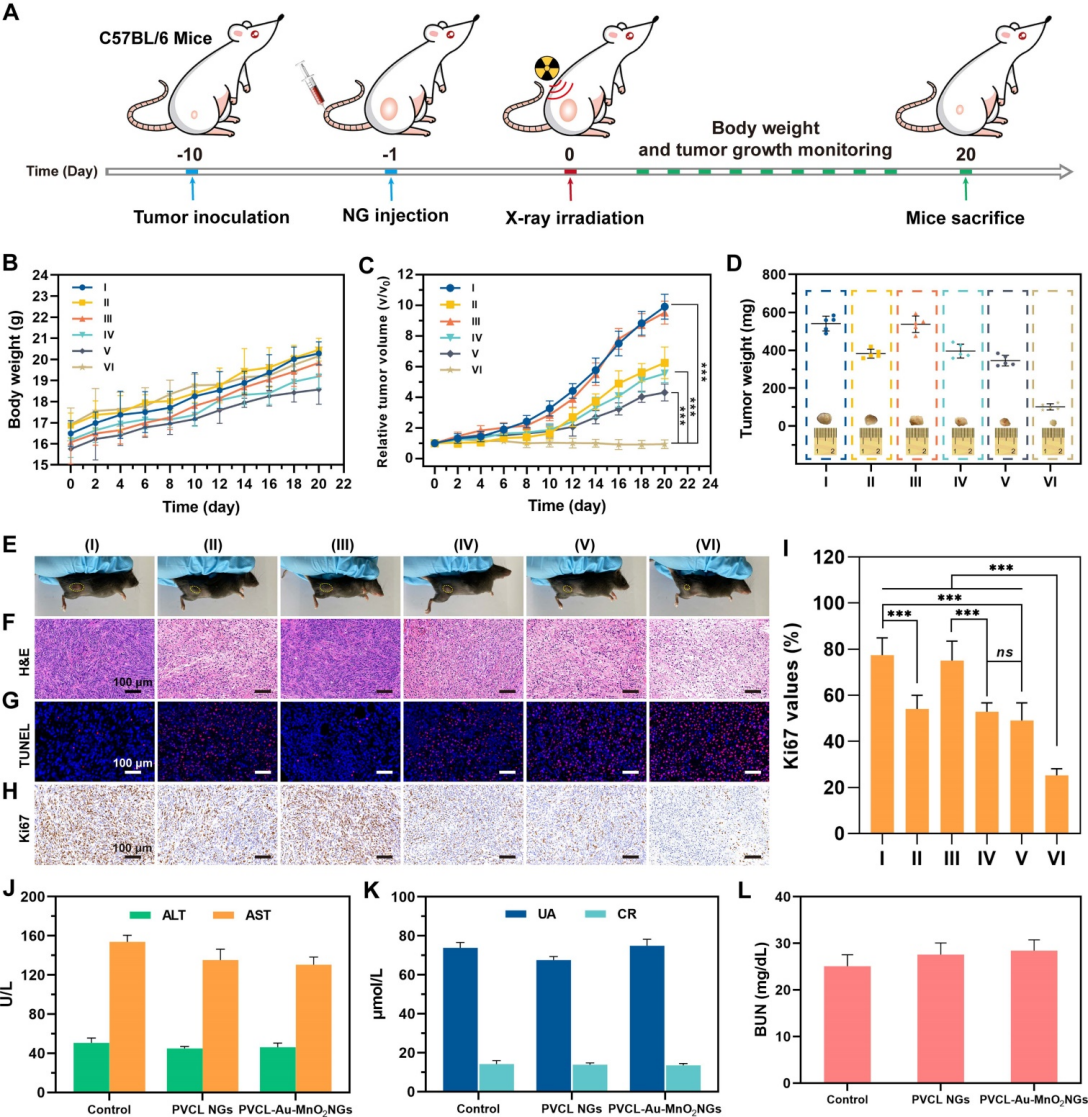
(A) Schematic illustration of the *in vivo* RT of tumor-bearing mice. (B) body weight and (C) tumor volume change of mice after different treatments. (D) Average tumor weight in different treatment groups (inset shows graphs of the excised tumor from each group). (E) Representative photographs of tumor-bearing mice in different treatment groups. (F) H&E-, (G) TUNEL- and (H) Ki67-staining photographs of tumor slices from different treatment groups. (I) Corresponding Ki67 values of tumor cells from the treatment groups in (H). (J, K and L) The blood biochemistry analyses of healthy female C57BL/6 mice at 7 days post i.v. injection of PBS, PVCL NGs or PVCL-Au-MnO_2_ NGs (45 mg mL^-1^, in 100 μL of PBS for each mouse), respectively (n = 3). The liver function was examined with alanine aminotransferase (ALT) and aspartate aminotransferase (AST), and the kidney function was examined with urea (UA), creatinine (CR) and blood urea nitrogen (BUN). The referred normal ranges for healthy mice from Servicebio, Inc. are as follows: ALT (10.06-96.47 U L^-1^), AST (36.3-235.48 U L^-1^), UA (44.42-224.77 μmol L^-1^), CR (10.91-85.09 μmol L^-1^) and BUN (10.81-34.74 mg dL^-1^), respectively. In (B-D, and I), n = 5 for each sample. *** represents *p* < 0.001, and *ns* indicates *p* > 0.05.

We then evaluated the therapeutic efficiency of PVCL-Au-MnO_2_ NGs for sensitized RT of tumors *in vivo* using a xenografted Pan02 tumor model (**Figure 7A**). The C57BL/6 mice bearing Pan02 tumors (~90 mm^3^) were divided into 6 groups (n = 6 for each group): (I) PBS control, (II) only RT (4 Gy), (III) PVCL NGs, (IV) PVCL NGs plus RT (4 Gy), (V) PVCL-Au-MnO_2_ NGs, and (VI) PVCL-Au-MnO_2_ NGs plus RT (4 Gy). The doses of groups III and IV were 45 mg mL^-1^ for NGs, and those of groups V and VI were 10 mM for Au, respectively, and all treatments were i.v. injection using 100 μL of PBS. As presented in **Figure 7B**, a regular body weight increase can be observed for all groups of treatments during the 20 days of treatment, indicating the low side effects of the NGs and X-ray radiation. As for the tumor inhibition studies (**Figure 7C**), the PVCL-Au-MnO_2_ NGs plus RT group (Group VI) induces the most significant tumor growth suppression among all groups. This superior inhibitory effect is likely owing to the enhanced radiosensitization effect enabled by PVCL-Au-MnO_2_ NGs, where both Au and MnO_2_ NPs play their respective significant roles. Additionally, the tumor growth in X-ray irradiation alone group (Group II), PVCL NGs plus X-ray irradiation group (Group IV) or PVCL-Au-MnO_2_ NGs group (Group V) display moderate tumor inhibition efficacy as compared with the PBS control or PVCL NGs groups (Group I or III). Notably, the antitumor efficacy of PVCL-Au-MnO_2_ NGs in the absence of RT may be ascribed to the CDT effect caused by ·OH generation. The excellent sensitized RT effect of PVCL-Au-MnO_2_ NGs plus X-ray radiation was also confirmed by the average tumor weights, and representative photographs of tumors and tumor-bearing mice in different experimental groups (**Figure 7D-E**).

To further evaluate the antitumor effect of PVCL-Au-MnO_2_ NGs with X-ray, one tumor tissue from each group was dissected at the 7th day post-treatment for hematoxylin and eosin (H&E), TdT-mediated dUTP Nick-End Labeling (TUNEL) and Ki67 staining. The H&E and TUNEL staining results (**Figure 7F-G** and **Figure S12**) indicate that the treatment of PVCL-Au-MnO_2_ NGs plus X-ray (Group VI) produces the maximum tumor cell necrosis and apoptosis effects among all groups. Meanwhile, as shown in **Figure 7H**, the tumor cell proliferation rate in Group VI is much lower than those in other groups, which was further confirmed by quantitative and statistical analysis of Ki67-stained cells (**Figure 7I**). Thus, the excellent sensitized RT effect of the PVCL-Au-MnO_2_ NGs may be attributed to a “full-process” radiosensitization mechanism, involveing in (i) converting H_2_O_2_ into ·OH and O_2_ under TME to induce cell cycle arrest at G2/M phase, thus improving cellular radiosensitivity prior to RT, (ii) concentrating radiation dose and amplifying the DNA damage during RT, as well as (iii) relieving the tumor hypoxia to inhibit DNA repair after RT. In addition, the H&E staining images show no noticeable damage of normal organs after different treatments (**Figure S13**), indicating the negligible side effect of PVCL-Au-MnO_2_ NGs and the applied X-ray. The blood hematological and serum biochemical markers (**Figure 7J-L** and **Figure S14**) of healthy mice in each treatment group are all within the normal ranges, further ensuring the good biosafety of PVCL-Au-MnO_2_ NGs to mice.

## Conclusion

In summary, we for the first time developed a multifunctional hybrid PVCL NG system incorporated with both Au and MnO_2_ NPs for a “full-process” sensitization of tumor RT. PVCL NGs formed through precipitation polymerization can be used as a nanoreactor to uniformly load Au NPs through fast NaBH_4_ reduction and MnO_2_ NPs through a mild reduction method. The formed PVCL-Au-MnO_2_ NGs with excellent colloidal and chemical stability display abilities to generate O_2_ and ·OH due to the incorporated MnO_2_ NPs that display the CAT-mimic activity and facilitate Mn^2+^-mediated Fenton-like reaction under TME, respectively. Meanwhile, these hybrid NGs can be taken up by cells, arrest cell cycle to the radiosensitive G2/M phase prior to RT, cause ROS-induced cell apoptosis through synergistic activation of caspases-, p53-, and DNA damage-mediated pathways during X-ray irradiation due to the incorporated Au NPs in the NGs that can localize the radiation dose, and prevent DNA repair due to the continuous production of O_2_ to relieve tumor hypoxia after RT. Furthermore, the PVCL-Au-MnO_2_ NGs could be used for dual-mode CT/MR imaging of tumors owing to the high X-ray attenuation capacity of Au NPs and the TME-triggered Mn^2+^ conversion from MnO_2_. Overall, we present an intelligent NG platform with TME-responsiveness for efficient tumor RT through a “full-process” radiosensitization, which may be extended for treatment of different tumor types.

## Experimental Section

### Synthesis of PVCL NGs

*N*-vinylcaprolactam (VCL) and AAEM were used as comonomers to synthesize PVCL NGs *via* a simple precipitation polymerization method according to the literature 39. In brief, VCL (469.5 mg, 3.4 mmol), N,N'-methylenebis(acrylamide) (14.5 mg, 0.094 mmol), and sodium dodecyl sulfate (8.8 mg, 0.03 mmol) were co-dissolved in 30 mL of water and stirred at 250 rpm. Half of the AAEM (42 mg, 0.196 mmol) dissolved in 5 mL of water was then dropwise added into the above solution. The mixture was purged with nitrogen and heated up to 70 ^o^C using a water bath under stirring for 30 min until a homogeneous solution was formed. Thereafter, the polymerization was initiated upon the addition of 2,2-azobis[N-(2-carboxyethyl)-2-methylpropionamidine] (11.7 mg mL^-1^, in 1.5 mL water). After initiation for 5 min, the other half of AAEM was added and the mixture was stirred at 70 ^o^C for another 4 h, resulting in a milky NG dispersion. After cooling down to room temperature, the dispersion was purified through dialysis against water *via* a membrane having a molecular weight cut-off (MWCO) of 8-14 kDa for five days to remove the unreacted monomers, followed by lyophilization to obtain the PVCL NGs as a white powder.

### Synthesis of PVCL-Au NGs

To incorporate Au NPs within the PVCL NGs, we adopted a literature protocol 39. In brief, freshly prepared aqueous solution of HAuCl_4_ (15 mg mL^-1^, 1 mL) was added to the PVCL NG suspension (5.6 mg mL^-1^, in 18 mL water) under magnetic stirring in ice bath for 1 h, leading to a light yellow Au(III)-PVCL NG complex suspension. After that, freshly prepared NaBH_4_ solution (22.5 mg mL^-1^, 2 mL in water) was quickly added to the reaction mixture under stirring. Immediately, the color of the solution changed from light yellow to deep purple, indicating the successful formation of Au NPs. The reaction solution was then stirred under an ice bath for another 12 h. The raw deep purple product was collected after removing the large Au particles through centrifugation (3000 rpm, 15 min). The supernatant was then purified through dialysis against water and lyophilized to obtain the PVCL-Au NGs as a purple powder according to the above protocols.

### Synthesis of PVCL-Au-MnO_2_ NGs

For *in-situ* incorporation of MnO_2_ NPs in the PVCL-Au NGs, a PVCL-Au NG dispersion (5 mg mL^-1^, 15 mL in water) was added with 1 mL of aqueous KMnO_4_ solution (6 mg mL^-1^) under stirring at room temperature overnight. The color of the reaction mixture slowly changed from deep purple to dark brown, indicating the successful formation MnO_2_ NPs, likely due to the fact that the β-diketone groups associated to AAEM containing in the NGs may act as a mild reductant to reduce MnO_4_^-^ to MnO_2_ NPs 39. The mixture was further purified and lyophilized in the same way as described above. For comparison, PVCL-MnO_2_ NGs without Au NPs were also prepared under the same experimental conditions.

### O_2_ generation capacity

Water was purged with pre-purified N_2_ for 30 min and adjusted to pH 6.5 before use. PVCL, PVCL-Au and PVCL-Au-MnO_2_ NGs were then separately dispersed in water (1 mg mL^-1^, 1 mL). Afterward, H_2_O_2_ was added to each solution to achieve a final concentration of 100 μM, and the O_2_ concentration was then recorded by an multiparameter benchtop meter (inoLab^®^Multi 9310 IDS, Xylem Analytics Germany Sales GmbH & Co. KG, Weilheim, Germany).

### *In vitro* cell culture assays

L929 cells (a mouse fibroblast cell line) were regularly cultured and adopted for cytotoxicity assay of different NGs. Pan02 cells (a mouse pancreatic adenocarcinoma cell line) were also regularly cultured and adopted for assays of cytotoxicity, cellular uptake, intracellular O_2_ and ROS generation, cell cycle arrest, colony formation, cell apoptosis, DNA damage and western blotting analysis after being treated with different NGs with or without X-ray irradiation.

### Animal experiments

All animal experiments were approved by the Animal Care and Use Committee of Donghua University, and were also performed in accordance with the guidance of the National Ministry of Health. Animal experiments including CT/MR imaging of tumors, *in vivo* pharmacokinetics and biodistribution of the hybrid NGs, *in vivo* antitumor therapeutic efficacy evaluation, histological examinations of tumors and major organs, and blood hematology and biochemistry were performed to examine the performances of tumor imaging and sensitized tumor RT after different treatments. See more details in the supplementary material.

## Supplementary Material

Supplementary methods and figures.
