# Supplementary material for: Intelligent design of polymer nanogels for full-process sensitized radiotherapy and dual-mode computed tomography/magnetic resonance imaging of tumors
Source: Theranostics. 2022 Apr 18;12(7):3420–37. doi: 10.7150/thno.70346 (PMC9065179; doi:10.7150/thno.70346)
Supplement: Supplementary file 1 — Supplementary methods and figures. [file thnov12p3420s1.pdf]

Supporting information

Intelligent design of polymer nanogels for full-process sensitized radiotherapy and dual-mode computed tomography/magnetic resonance imaging of tumors

Changchang Zhang[1, ‡], Wenzhi Tu[2, ‡], Xuming Chen[2], Bing Xu[2], Xin Li[3,4], Chaolei Hu[3,4], Mingwu Shen[1], Shaoli Song[5], Chunjuan Jiang[5], Shengyu Yao[2], Andrij Pich[,3,4], Yong Liu*[,2], Xiangyang Shi*[,1]*

[1] State Key Laboratory for Modification of Chemical Fibers and Polymer Materials, Shanghai Engineering Research Center of Nano-Biomaterials and Regenerative Medicine, College of Chemistry, Chemical Engineering and Biotechnology, Donghua University, Shanghai 201620, P. R. China

[2] Department of Radiation Oncology, Shanghai General Hospital, Shanghai Jiao Tong University School of Medicine, Shanghai 200080, P. R. China

[3] DWI-Leibniz-Institute for Interactive Materials e.V., Forckenbeckstr. 50, 52056, Aachen, Germany

[4] Functional and Interactive Polymers, Institute of Technical and Macromolecular Chemistry, RWTH Aachen University, Worringerweg 2, 52074, Aachen, Germany

[5] Department of Nuclear Medicine, Shanghai Cancer Center, Fudan University, Shanghai 200030, P. R. China

---

* Corresponding author. E-mail: pich@dwi.rwth-aachen.de (A. Pich), yong.liu2@shgh.cn (Y. Liu), and xshi@dhu.edu.cn (X. Shi)

‡ Authors contributed equally to this work.

Experimental Section

Materials. *N*-Vinylcaprolactam (VCL), N,N'-methylenebis(acrylamide) (BIS), sodium borohydride ($NaBH_4$) and potassium permanganate ($KMnO_4$) were purchased from J&K Scientific Ltd. (Shanghai, China). Acetoacetoxyethyl methacrylate (AAEM) was from TCI Development Co., Ltd. (Shanghai, China). 2,2-Azobis[N-(2-carboxyethyl)-2-methylpropionamidine] (ACMA) was obtained from Wako Pure Chemical Industries, Ltd. (Osaka, Japan). Gold(III) chloride trihydrate ($HAuCl_4 \cdot 3H_2O$) and all the other chemicals were acquired from Sinopharm Chemical Reagent Co., Ltd. (Shanghai, China). Cell Counting Kit-8 (CCK-8) was from Beyotime Institute of Biotechnology (Shanghai, China). Fetal bovine serum (FBS) was from Gibco Life Technologies Co. (Grand Island, NY). Dulbecco's modified Eagle's medium (DMEM), RPMI 1640 medium, penicillin-streptomycin and trypsin were obtained from HyClone Lab., Inc. (Logan, UT). Regenerated cellulose dialysis membranes with a molecular weight cut-off (MWCO) of 8-14 KDa were acquired from Fisher (Pittsburgh, PA). Water used in all experiments was purified by a RephiLe purist UV ultrapure water system with a resistivity higher than 18.2 MΩ.cm (RephiLe Bioscience, Ltd., Shanghai, China).

Characterization techniques. The size and morphology of the obtained PVCL NGs, PVCL-Au NGs and PVCL-Au-MnO_2 NGs were analyzed by field emission scanning electron microscope (FE-SEM, S-4800, Hitachi, Ltd., Tokyo, Japan) at a voltage of 15 kV or transmission electron microscopy (TEM, JEM-2100F, JEOL Ltd., Tokyo, Japan) under an accelerating voltage of 200 kV. The sample for SEM analysis was prepared by dropping an NG suspension (500 μg mL^{-1}, 10 μL in acetone) onto the silicon wafer, followed by air drying and sputter coating of a gold film with a thickness of 10 nm. For TEM analysis, PVCL-Au or PVCL-Au-MnO_2 NGs (500 μg mL^{-1}, 10 μL in acetone) were deposited onto a carbon-coated copper grid and air-dried before measurements. The composition of hybrid NGs was determined using X-ray elemental mapping techniques (OXFORD INCA ENERGY 350, Oxford Instruments, Abingdon, UK) attached to the TEM instrument. The samples were analyzed by X-ray photoelectron spectroscopy (XPS, ESCALab250, Thermo Fisher Scientific Inc., Eugene, OR). Hydrodynamic size distribution and ζ-potential of the synthesized PVCL, PVCL-Au and PVCL-

Au-MnO_2 NGs were measured using a Malvern zetasizer (Nano ZS model ZEN3600 with a standard 633 nm laser, Worcestershire, UK). Au and MnO_2 contents in the PVCL-Au-MnO_2 NGs were determined by a Leeman Prodigy inductively coupled plasma-optical emission spectroscopy (ICP-OES, Hudson, NH). The samples were digested by *aqua regia* and diluted with water before measurements.

Tumor microenvironment (TME)-responsive Mn^{2+} release. To test the pH- and GSH-responsive Mn^{2+} release, PVCL-Au-MnO_2 NGs (5 mg) were dispersed in 1 mL of phosphate buffer containing 100 μM of H_2O_2 under the following conditions: (1) pH = 7.4, (2) pH = 6.5, (3) pH = 7.4 + GSH (10 mM), or (4) pH = 6.5 + GSH (10 mM). Each solution was put in the dialysis bag (MWCO = 8-14 KDa), and then submerged into 9 mL of the corresponding buffer. Then, 1 mL of the outer-phase buffer medium was pipetted out at each scheduled time interval. The Mn^{2+} contents were determined using a Spectroquant® Mn^{2+} test kit (Merck KGaA, Darmstadt, Germany) according to the manufacturer's instruction.

·OH generation by Mn^{2+}-mediated Fenton-like reaction. First, we used $MnCl_2$ as a model to evaluate the Mn^{2+}-mediated Fenton-like reaction according to the literature [1]. In brief, methylene blue (MB) was used as a substrate (10 μg mL^{-1}) to test the ·OH generation ability. In the presence of H_2O_2 (8 mM) dissolved in 25 mM $NaHCO_3$/5% CO_2 buffer solution (5 mL), $MnCl_2$ was added to achieve a final concentration of 0.5 mM. The reaction was performed at 37 °C for 30 min and the changes of MB absorbance indicating the ·OH-induced MB degradation in the mixture solution at 665 nm were monitored using a Pekin-Elmer Lambda 25 UV-vis spectrophotometer (Boston, MA).

To check the concentration-dependent ·OH generation capacity, H_2O_2 or Mn^{2+} with different concentrations were dissolved in 25 mM $NaHCO_3$/5% CO_2 buffer solution containing 10 μg mL^{-1} MB. At a fixed $MnCl_2$ concentration (0.5 mM), H_2O_2 with different concentrations (0, 1, 2, 4, 6, 8 or 12 mM) was incubated with the buffer at 37 °C for 30 min. Meanwhile, at the fixed H_2O_2 concentration of 8 mM, the effect of Mn^{2+} concentration (0, 0.1, 0.2, 0.3, 0.4, 0.5, 0.8 or 1.0 mM) on the ·OH generation in the 25 mM $NaHCO_3$/5% CO_2 buffer solution containing 10 μg mL^{-1} MB was also studied.

All reactions were monitored by the absorbance change at 665 nm through UV-vis spectrometry.

Lastly, under the optimized Mn^{2+}-mediated Fenton-like reaction condition (0.5 mM Mn^{2+} and 8 mM H_2O_2), we examined the ·OH generation of PVCL-Au-MnO_2 NGs in the presence of GSH and the effect of GSH on the ·OH scavenging. PVCL-Au-MnO_2 NGs ([Mn] = 0.5 mM) were incubated with 25 mM $NaHCO_3$/5% CO_2 buffer solution containing GSH with different concentrations (0, 0.5, 1, 2, 5 or 10 mM), 10 μg mL^{-1} MB, and 8 mM H_2O_2 at 37 °C for 30 min. Each mixture solution was monitored by UV-vis spectrometry to record the MB absorbance change at 665 nm. For comparison, $MnCl_2$ (0.5 mM) was also tested under the same conditions.

Electron spin resonance (ESR) measurements. A high-performance benchtop ESR instrument (Bruker EMXnano, Karlsruhe, Germany) was used to detect the ·OH using 5,5-dimethyl-1-pyrroline N-oxide as a trapping agent. PVCL-Au-MnO_2 NGs ([Mn] = 0.5 mM), H_2O_2 (8 mM) and GSH (5 mM) were dispersed or dissolved in 25 mM $NaHCO_3$/5% CO_2 buffer solution. At time points of 0, 5, 10, and 30 min, ESR spectra of the above mixture were recorded. The following instrument settings were used to collect ESR spectra: 0.1 G field modulation, 100 G scan range, and 30 s sweep time.

X-ray attenuation property. The X-ray attenuation property of PVCL-Au-MnO_2 NGs was studied using a micro-CT system (SIEMENS Inveon MM, Erlangen, Germany) at 70 kV and a slice thickness of 0.1 mm. PVCL-Au-MnO_2 NGs with different Au concentrations were tested, and the X-ray attenuation intensity was determined in Hounsfield units (HU).

TME-responsive magnetic resonance (MR) relaxometry. For the T_1 MR relaxometry study, a 0.5 T NMR analyzing and imaging system (NMI20, Niumag, Shanghai, China) was used to measure the T_1 relaxation times. PVCL-Au-MnO_2 NGs at different Mn concentrations ([Mn] = 0.0625, 0.125, 0.25, 0.5 or 1.0 mM) were dispersed in phosphate buffer (0.5 mL) containing H_2O_2 (100 μM) under the following conditions: (1) pH = 7.4, (2) pH = 6.5, (3) pH = 7.4 + GSH (10 mM), or (4) pH = 6.5 + GSH (10 mM). The mixture was incubated at room temperature for 30 min, and then the T_1 relaxation time of each sample was measured under the follow parameters: IR sequence, Echo time (TE) = 20.0 ms, repetition time (TR) = 300.0 ms, slice thickness = 0.8 mm, and field of view (FOV) = 108 × 108

mm. T_1 relaxivity (r_1) was calculated by fitting the plot of inverse T_1 relaxation time ($1/T_1$) as a function of Mn concentration. The MR phantom images of PVCL-Au-MnO_2 NGs under various conditions were obtained using a clinical MR imaging system (Bruker Biospec 7T micro-MR imaging system, Karlsruhe, Germany). The detailed parameters were set as follows: TE = 6.0 ms, TR = 1000.0 ms, slice thickness = 1.0 mm, and FOV = 65 × 85 mm.

Cell culture. Pan02 cells (a mouse pancreatic adenocarcinoma cell line) were obtained from China Infrastructure of Cell Line Resource and cultured in DMEM containing 5% FBS and 1% penicillin-streptomycin. L929 cells (a mouse fibroblast cell line) were obtained from Institute of Biochemistry and Cell Biology (the Chinese Academy of Sciences, Shanghai, China) and cultured in RPMI 1640 medium containing 10% FBS and 1% penicillin-streptomycin.

Hemolysis, cytotoxicity, and cellular uptake assays *in vitro*. Hemolysis assay was performed to evaluate the hemocompatibility of the PVCL-Au-MnO_2 NGs according to the literature [2]. In brief, 1.5 mL of blood collected from the inner canthus vein plexus of mice was diluted with 3.5 mL of phosphate buffered saline (PBS), and then the pure red blood cells (RBCs) were obtained *via* repeated centrifugation/redispersion processes (2000 rpm, 10 min, 3 times). The RBCs were then diluted with 5 mL of PBS. Thereafter, 100 μL of the obtained RBC suspension was mixed with 900 μL water (positive control), PBS (negative control) and PVCL-Au-MnO_2 NG dispersed in PBS at various concentrations (12.5-400 μg mL^{-1}). After 2 h incubation at 37 ºC, each sample was centrifuged at 13000 rpm for 15 min. UV-vis spectrometry was used to record the absorbance of the supernatant at 540 nm.

For cytotoxicity assays, L929 or Pan02 cells were seeded into 96-well plates at a density of 1×10^4 cells per well in 5% CO_2 at 37 ºC overnight to allow the attachment of cells. The next day, the medium of each well was replaced with 100 μL complete medium containing PVCL NGs, PVCL-Au NGs, PVCL-MnO_2 NGs or PVCL-Au-MnO_2 NGs at different NG concentrations (0, 6.25, 12.5, 25, 50, 100, 200, 300 and 400 μg mL^{-1}, respectively). For L929 cells, RPMI 1640 medium was used, while Pan02 cells were cultured using DMEM. The cells were incubated for additional 24 h. After that, 100 μL medium containing 10% CCK-8 agent was added to each well, and cells were incubated under

regular culture conditions for 4 h. Then, the optical density of each well was examined using a Multiskan MK3 ELISA reader (Thermo Scientific, Waltham, MA).

To examine the cellular uptake of the PVCL-Au-MnO_2 NGs, cells treated with NGs were observed by TEM. Pan02 cells were seeded into 6-well plates at a density of 2 × 10^5 cells per well overnight. Then, the cells were treated with fresh complete DMEM containing PVCL-Au-MnO_2 NGs (200 μg mL^{-1}) for 12 h at 37 ºC. Then, the cells were washed with PBS for three times, fixed with 1 mL of glutaraldehyde (2.5%) at room temperature for 30 min, washed with PBS for three times, collected with cell scraper and fixed again with 1 mL of glutaraldehyde (2.5%) at 4 ºC for 12 h. Then, each cell sample was processed under standard protocols before TEM observation using a Hitachi TEM system (Tokyo, Japan) with an operation voltage of 80.0 kV. To quantify the cellular uptake of NGs, the cells were incubated with PVCL-Au-MnO_2 NGs (200 μg mL^{-1}) for 3, 6, and 12 h, respectively according to the above protocols. Then, the cells were washed with PBS thrice, trypsinized, collected through centrifugation (1000 rpm, 5 min), and counted. Afterward, the cells were digested by *aqua regia* for 4 h, and diluted with water before measurements of the Au or Mn content by ICP-OES.

Intracellular O_2 generation. Pan02 cells were seeded into the glass-bottom cell culture dishes with a diameter of 15 mm (Wuxi NEST Biotechnology Co., Ltd., Wuxi, China) at a density of 1 × 10^5 cells per dish (1 mL DMEM) and cultured overnight. Then, the medium of each well was replaced with complete DMEM containing $[Ru(dpp)_3]Cl_2$ at a concentration of 10 μg mL^{-1}, and the cells were regularly cultured for 4 h. After being carefully washed with PBS for three times, cells were incubated with complete DMEM containing PVCL, PVCL-Au, PVCL-MnO_2 or PVCL-Au-MnO_2 NGs (200 μg mL^{-1}) for 12 h under 37 ºC and 5% CO_2. Cells treated with PBS (100 μL) were set as control. Then, the cells were washed and the intracellular luminescence of $[Ru(dpp)_3]Cl_2$ was observed using a ZEISS LSM 700 confocal microscope (Jena, Germany).

Intracellular ·OH production. DCFH-DA reactive oxygen species (ROS) assay kit was utilized to monitor the intracellular ROS generation. Pan02 cells were cultured according to the procedures described above (see Intracellular O_2 generation). After overnight culture, fresh complete DMEM

containing PVCL, PVCL-Au, PVCL-MnO_2 or PVCL-Au-MnO_2 NGs (200 μg mL^{-1}) was added to each well of cells, which were further cultured for additional 12 h. Cells treated with PBS (100 μL) were set as control. After washing the cells with PBS twice, DCFH-DA (10 μM) was added to each well of cells and the cells were incubated for another 30 min before confocal microscopic imaging of the intracellular green fluorescence of cells.

Cell cycle analysis. Pan02 cells were seeded into 6-well plates at a density of 2 × 10^5 cells per well overnight. Then, the cells in each well were incubated with 2 mL of complete DMEM containing 200 μg mL^{-1} of PVCL, PVCL-Au, PVCL-MnO_2, or PVCL-Au-MnO_2 NGs for 12 h. Cells treated with PBS (200 μL) were set as control. After that, cells in each well were washed with PBS thrice, trypsinized, collected through centrifugation (1000 rpm, 5 min), and fixed with 70% precooled ethanol at 4 °C for 12 h. After that, the cells were washed with PBS thrice and stained with a mixture solution containing 1% Triton X-100, 0.01% RNase and 0.05% propidium iodide (PI) for 30 min at 37 °C in the dark. Flow cytometry was employed to quantify the DNA content to estimate the percentages of the cell population in different phases of each cell cycle. Modfit software (Verity Software House, Topsham, ME) was used for fitting analysis.

Clonogenic assay. Pan02 cells were cultured and treated with NGs under the same conditions described above for cell cycle analysis except the cell seeding density (5 × 10^5 cells per well). Then, the cells in each group were washed twice with PBS and received the X-ray irradiation at 0, 2, 4, 6 and 8 Gy, respectively. Next, depending on the different radiation dose (0, 2, 4, 6 or 8 Gy), the cells in each group were digested, counted, and seeded at a density of 200, 400, 800, 2000, or 4000 cells per well in 6-well plates, followed by a further incubation of 10 days. For the X-ray irradiation treatment, the total exposure irradiation time of cells during the procedure was 1.1 min. The irradiation equipment was the medical linear accelerator (Varian Clinac IX), which used 6 MV X-ray, and the size of the radiation field was 30 × 30 cm. Before irradiation, we used two equivalent solid water plates with a thickness of 5 mm to simulate the cell fluid, and the calibrated MOSFET detector was placed between these two solid water plates. Then, the accelerator with an output of 500 monitor unit (MU) was used,

and the absorbed dose of the detector was recorded. On this basis, the accelerator MU value corresponding to the irradiation dose to the cells was calculated, and the absorption coefficient was also read by the detector. Colonies were stained with 1.0% crystal violet and counted to evaluate the effect of the respective treatment. The surviving fraction of each group was measured in triplicate through the formula of surviving fraction = (surviving colonies)/(cells seeded × plating efficiency). The classical multitarget single-hit model was adopted to perform the nonlinear fitting of the cell survival fraction of each group using Graphpad prism® 8.0 software (GraphPad Software Inc., San Diego, CA). Meanwhile, the sensitization enhancement ratio (SER) of each group was also calculated *via* the multitarget single-hit model.

Apoptosis assay. Pan02 cells were seeded in 6-well plates at a density of 5×10^5 cell per well (2 mL DMEM) overnight. Then, the cells were incubated with complete DMEM containing PVCL-Au, PVCL-MnO_2, or PVCL-Au-MnO_2 NGs (NG concentration = 200 μg mL^{-1}) for 12 h. Cells treated with PBS (200 μL) were used as control. After that, the cells were exposed to X-ray irradiation (4 Gy, 3.6 Gy min^{-1}), followed by further incubation for 24 h. Subsequently, the cells in each well were trypsinized, stained with Annexin V-Fluorescein Isothiocyanate/PI Kit according to the manufacturer's instruction, and analyzed by a Becton Dickinson Accuri C6 Flow Cytometer (Bedford, MA). Each sample was measured for three times.

Detection of ROS after X-ray radiation. Pan02 cells were seeded in 12-well plates at a density of 1×10^5 cell per well (1 mL DMEM) for 24 h. Then, the cells were incubated with complete DMEM containing PVCL-Au, PVCL-MnO_2, or PVCL-Au-MnO_2 NGs (200 μg mL^{-1}) for 12 h. After that, the cells were exposed to X-ray irradiation (4 Gy, 3.6 Gy min^{-1}), followed by further incubation for 1 h. Cells treated with PBS only (100 μL) were set as control. Subsequently, the cells were washed twice with PBS, and stained with DCFH-DA for 30 min at 37 °C and 5% CO_2. After the washing and trypsinization process, the fluorescence signals of cells were detected using flow cytometry. For each sample, the mean fluorescence intensity of 1×10^4 cells was recorded to determine the intracellular content of ROS and each measurement was repeated for three times.

Western blotting analysis. Pan02 cells were seeded in 6-well plates at a density of 5 × 10^5 cells per well (2 mL DMEM) for 24 h. The cells were then incubated with complete DMEM containing PVCL-Au-MnO_2 NGs (200 μg mL^{-1}) for 12 h, followed by washing twice with PBS before the treatment of X-ray irradiation (4 Gy, 3.6 Gy min^{-1}). After that, the cells were lysed and the effects of NG treatment with or without X-ray on the expression levels of the related proteins were determined by western blotting according to our previous work [3]. Cells treated with PBS (with or without X-ray radiation) were also analyzed for comparison.

DNA damage assay. Pan02 cells were seeded into the glass bottom cell culture dishes (diameter = 15 mm) at a density of 1 × 10^5 cells per dish (1 mL DMEM) and cultured overnight. The next day, cells were incubated with complete DMEM containing PVCL, PVCL-Au, PVCL-MnO_2, or PVCL-Au-MnO_2 NGs (200 μg mL^{-1}) for 12 h. Next, the cells were subjected to 4 Gy X-ray irradiation (3.6 Gy min^{-1}). After another 0, 1 and 24 h, the cells were washed twice with PBS and fixed with 4% paraformaldehyde for 30 min, followed by the treatment of 1% Triton X-100 to enhance the cell permeabilization. Then, the cells were incubated with phospho-histone H2AX (γ-H2AX) antibody (Cell Signaling Technology, Inc., Shanghai, China) at 4 °C overnight after being treated with 1% bovine serum albumin for 1 h. After that, the cells were treated with goat anti-rabbit IgG H&L (Alexa Fluor® 448) for 1 h, and the cell nuclei were stained by DAPI. Finally, the cells were observed by confocal laser scanning microscopy (Leica, Wetzlar, Germany). Cells treated with PBS (100 μL) were used as control.

Animals and tumor model. All animal experiments were approved by the Animal Care and Use Committee (IACUC) of Donghua University, and were also performed in accordance with the guidance of the National Ministry of Health. Female C57BL/6 mice with 4-6 weeks old (body weight of 15-20 g) were obtained from the Shanghai Slac Laboratory Animal Center (Shanghai, China). The xenograft tumor model was established by injection of 100 μL PBS solution of Pan02 cells (2 × 10^6 cells) into the right thigh of each C57BL/6 mouse.

Pharmacokinetics and biodistribution. To explore the pharmacokinetics of PVCL-Au-MnO_2

NGs *in vivo*, healthy C57BL/6 mice were intravenously (i.v.) administrated with PVCL-Au-MnO_2 NGs ([Au] = 10 mM, in 100 μL of PBS for each mouse). The blood samples at various time points (0.5, 1, 2, 4, 8, 12, 24, and 48 h, respectively) were collected, weighed, and digested by *aqua regia* for 24 h. Each sample was diluted with water, and the Au content was quantified by ICP-OES. The blood circulation half-decay time was determined using a one-phase decay exponential model by Graphpad prism® 8.0 software.

To study the biodistribution of the NGs, the tumor-bearing mice were i.v. administrated with PVCL-Au-MnO_2 NGs ([Au] = 10 mM, in 100 μL of PBS for each mouse). At 2, 6, 12, 24 or 48 h post-injection, the mice were sacrificed. Major organs (heart, liver, spleen, lung and kidney) and tumors were dissected, rinsed with PBS, weighed, and digested by *aqua regia* for 5 days. The biodistribution of PVCL-Au-MnO_2 NGs in tumors and different organs were calculated as the mass of Au per gram of tissues based on ICP-OES quantification.

***In vivo* dual-mode CT/MR imaging.** For *in vivo* CT imaging, PVCL-Au-MnO_2 NGs ([Au] = 10 mM, in 100 μL of PBS) were i.v. injected to each tumor-bearing C57BL/6 mouse. Each mouse was then anesthetized by pentobarbital sodium solution (1%, w/w, 4 μL g^{-1}). CT scanning was performed using the micro-CT system mentioned above under the same instrumental conditions before and at 24 h post-injection. For *in vivo* MR imaging, PVCL-Au-MnO_2 NGs ([Mn] = 10 mM, in 100 μL of PBS) were i.v. injected to each tumor-bearing C57BL/6 mouse. Then, MR scanning was performed using the Bruker Biospec 7T micro-MR imaging system as mentioned above. The detailed parameters were set as follows: TE = 11.7 ms, TR = 1658.0 ms, and slice thickness = 0.5 mm. MR images before and at 24 h post-injection were acquired, and MR signal to noise ratio (SNR) was quantified using the signal of air as the noise.

***In vivo* radiotherapy (RT) of tumors.** C57BL/6 mice bearing Pan02 tumors (~90 mm^3) were divided into six groups (n = 6 for each group): (I) PBS control (100 μL PBS), (II) only RT (100 μL PBS, 4 Gy), (III) PVCL NGs (i.v. injection, 45 mg mL^{-1}, in 100 μL PBS), (IV) PVCL NGs (i.v. injection, 45 mg mL^{-1}, in 100 μL of PBS) plus RT (4 Gy), (V) PVCL-Au-MnO_2 NGs (i.v. injection, [Au] = 10 mM, in 100 μL of PBS), and (VI) PVCL-Au-MnO_2 NGs (i.v. injection, [Au] = 10 mM, in

100 μL of PBS) plus RT (4 Gy). At 24 h post i.v. injection of NGs, tumors in groups (II), (IV) and (VI) were subjected to X-ray irradiation. The mouse weight and tumor size (length and width) were monitored every two days for 20 days. The tumor volumes were calculated by the following formula: volume = length × width^2/2.

Tumor hypoxia relief evaluation. The tumor hypoxia was evaluated using a Hypoxyprobe™ Green Kit according to the literature [4]. C57BL/6 mice bearing Pan02 tumors were divided into five groups: (1) control, (2) PVCL NGs, (3) PVCL-Au NGs, (4) PVCL-MnO_2 NGs, and (5) PVCL-Au-MnO_2 NGs ([Mn] = 10 mM for all MnO_2-associated groups, concentrations of PVCL NGs and PVCL-Au NGs are equivalent to that of PVCL-Au-MnO_2 NGs). At 24 h post-injection, the mouse in each group was i.v. administrated with pimonidazole hydrochloride (Hypoxyprobe™-1, 60 mg kg^{-1}). The tumors were excised at 2 h post-treatment, stored in liquid nitrogen and fixed under hypothermia. After that, the frozen tumor sections were immunostained with FITC-MAb1 and observed using the optical microscope.

Blood hematology and biochemistry analysis. Healthy C57BL/6 mice (n = 3) were i.v. injected with 100 μL of PVCL or PVCL-Au-MnO_2 NGs (in PBS, 45 mg mL^{-1}), respectively. The mice treated with PBS were set as the blank control. At 7 days post-treatment, blood was harvested from the mice in each group. About 500 μL of the blood from each mouse in each group was collected in the anticoagulant tube containing heparin for hematology analysis. Another 500 μL blood sample from each mouse in each group was collected in the sterile centrifuge tube, set at room temperature for 1 h, and centrifuged at 2000 rpm for 20 min to obtain the blood serum. The parameters of blood hematology and biochemistry including white blood cell (WBC), RBC, platelets (PLT), hemoglobin (HGB), hematocrit (HCT), mean corpuscular hemoglobin concentration (MCHC), alanine aminotransferase (ALT), aspartate aminotransferase (AST), urea (UA), creatinine (CR), and blood urea nitrogen (BUN) were analyzed by Servicebio Technology Co., Ltd. (Wuhan, China).

Histological examinations. For histological analysis, one tumor tissue at 7 days post-treatment in each group was collected, and the major organs (heart, liver, spleen, lung and kidney) at 20 days

post-treatment in each group were harvested, fixed in 4% paraformaldehyde, embedded in paraffin, and sectioned through standard protocols [5]. The pathological and morphological changes of the tumor tissues and major organs were studied by hematoxylin and eosin (H&E) staining. Additionally, the tumor cell proliferation and apoptosis were analyzed by immunohistochemical staining of proliferating cell nuclear antigen Ki67 monoclonal antibody and immunofluorescence staining with TdT-mediated dUTP Nick-End Labeling (TUNEL), respectively.

Statistical analysis. All experimental data were given as the mean ± standard deviation ($n \geq 3$). Scientific graphing, comprehensive curve fitting, and data organization were performed by GraphPad Prism® 8.0 software. A p value of 0.05 was selected as the significance level, and all data were marked as (*ns*) for $p > 0.05$, (*) for $p < 0.05$, (**) for $p < 0.01$, and (***) for $p < 0.001$, respectively.

Table S1. The MnO_2 loading efficiency (LE) and loading content (LC) in the PVCL-Au-MnO_2 NGs with different $KMnO_4$/Au feeding mass ratios.

Feeding mass ratio ($KMnO_4$/Au)	LE	LC
0.5:1	98.7%	1.1%
1:1	98.2%	2.1%
2:1	89.1%	3.7%
4:1	53.4%	4.5%

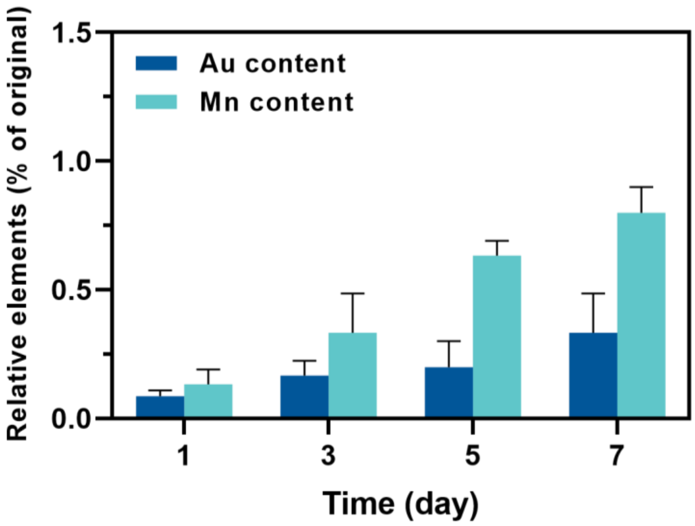

Figure S1. Relative Au and Mn contents (% of original) in the supernatants of PVCL-Au-MnO_2 NGs (1 mg mL^{-1}) after storage (in PBS, pH 7.4, at 37 °C for 1, 3, 5 and 7 days, respectively) and centrifugation (12000 rpm, 15 min). The Au and Mn contents were determined by ICP-OES. The data are shown as mean ± SD (n = 3).

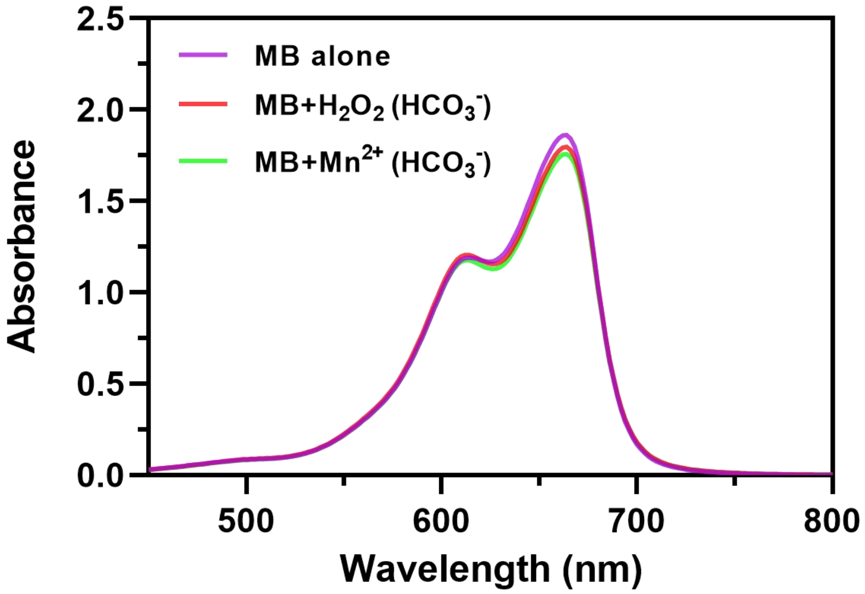

Figure S2. UV-vis spectra of MB (10 μg mL^{-1}) after treatment with H_2O_2 (8 mM) or $MnCl_2$ (0.5 mM) alone in the buffer of 25 mM $NaHCO_3$/5% CO_2.

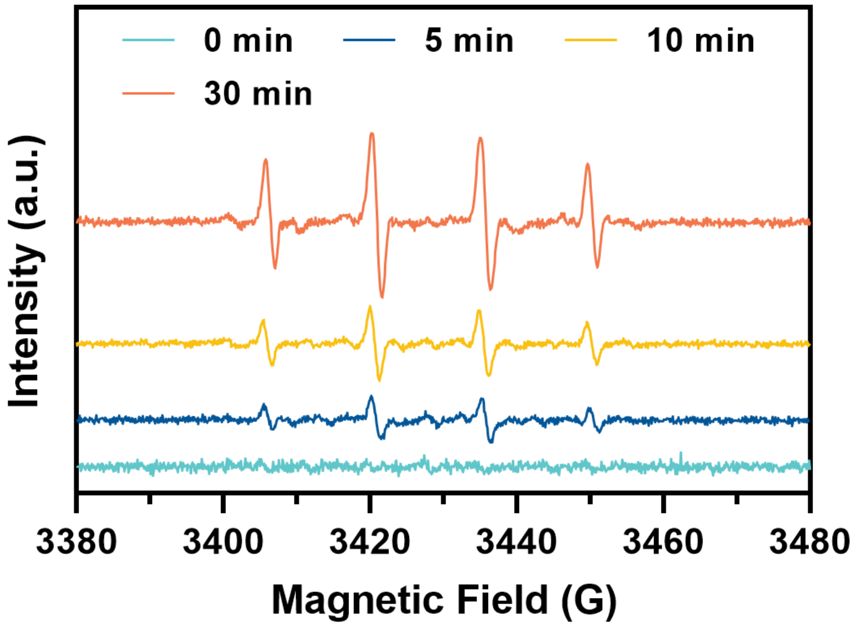

Figure S3. ESR spectra of ·OH collected at different time points from the reaction system containing PVCL-Au-MnO_2 NGs ([Mn] = 0.5 mM), 8 mM H_2O_2, 5 mM GSH, and 25 mM $NaHCO_3$/5% CO_2.

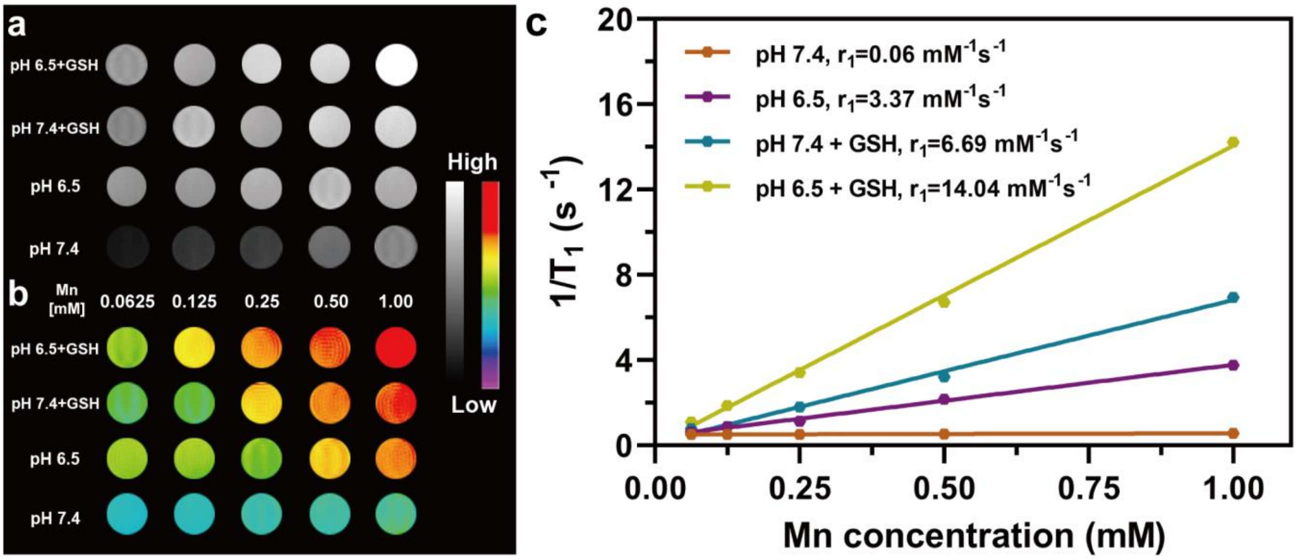

Figure S4. (a) T_1-weighted MR images, (b) pseudocolor MR images and (c) the plot of $1/T_1$ (as a function of Mn concentration) of PVCL-Au-MnO_2 NG dispersion containing H_2O_2 (100 μM) at different pHs (6.5 and 7.4) with or without GSH (10 mM).

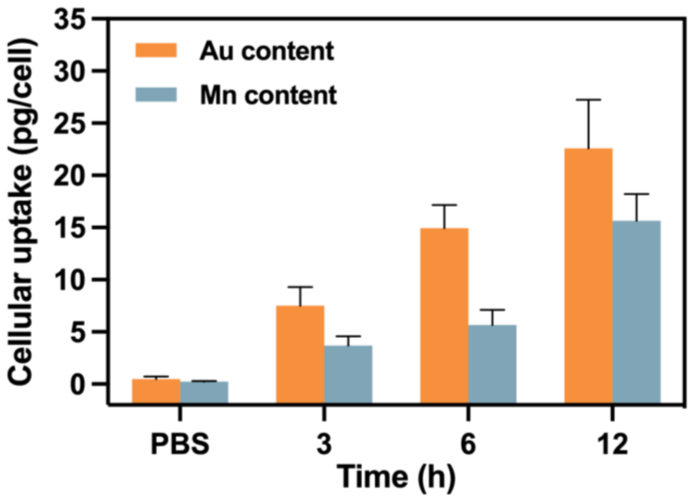

Figure S5. Au and Mn uptake in Pan02 cells treated with PVCL-Au-MnO_2 NGs (200 μg mL^{-1}) for 3, 6 or 12 h. Pan02 cells treated with PBS were used as control. The data are shown as mean ± SD (n = 3).

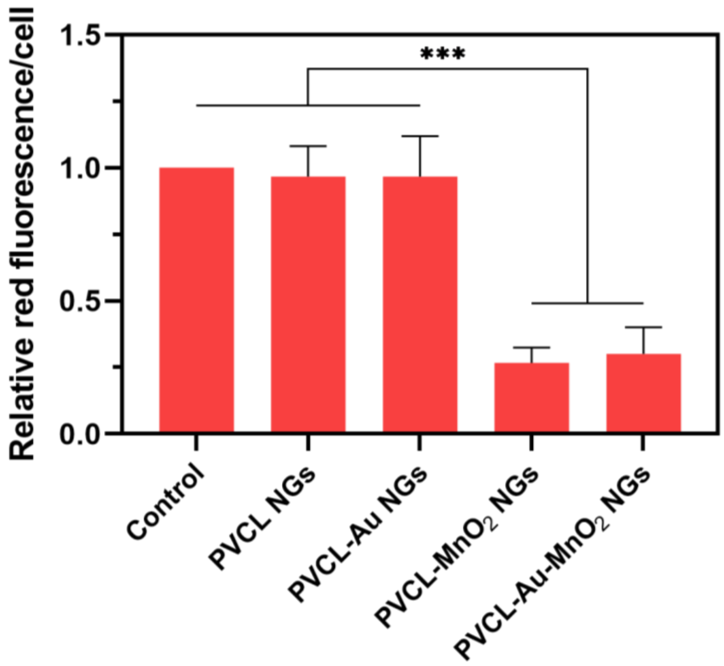

Figure S6. Quantitative analysis of O_2 level in Pan02 cells after different treatments at an NG concentration of 200 μg mL^{-1}.

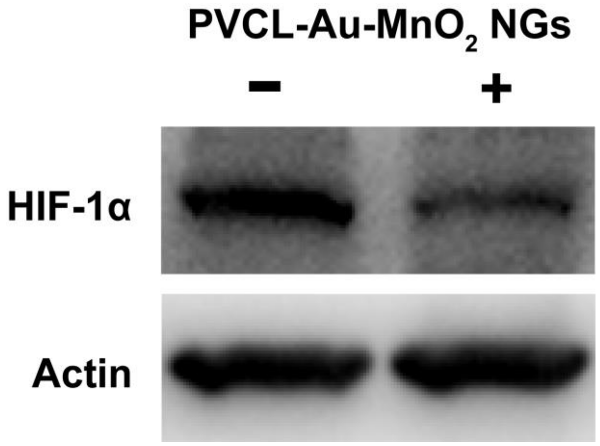

Figure S7. Hypoxia-inducible factor (HIF-1α) expression levels of Pan02 cells before (-) and after (+) being treated with PVCL-Au-MnO_2 NGs (200 μg mL^{-1}) for 12 h.

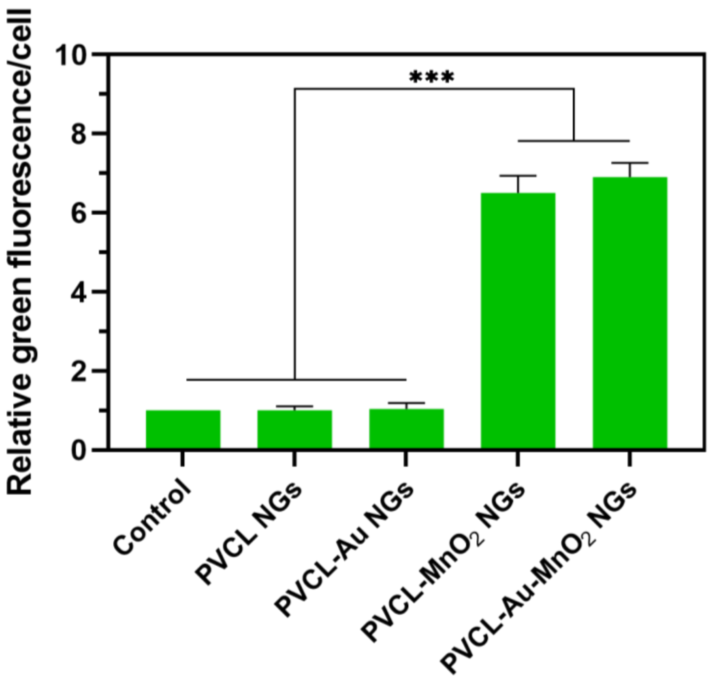

Figure S8. Quantitative analysis of ROS level in Pan02 cells after different treatments at an NG concentration of 200 μg mL^{-1}.

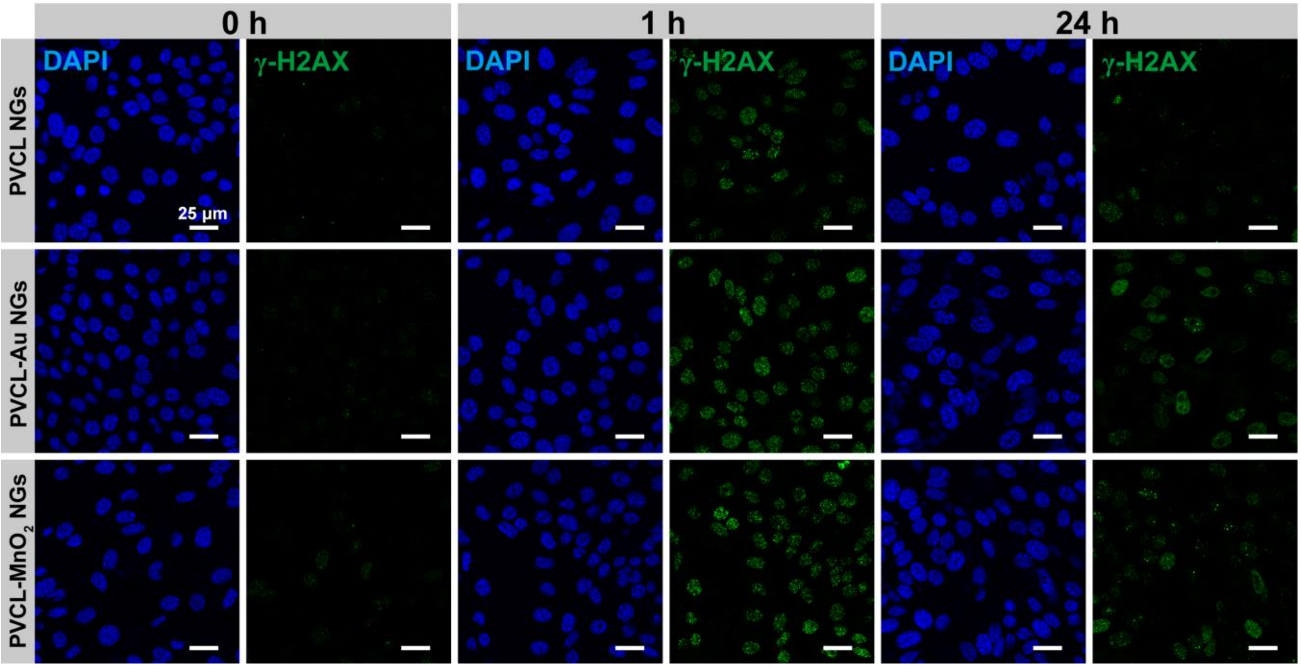

Figure S9. Changes of γ-H2AX foci (green) in cell nuclei (blue) of Pan02 cells treated by PVCL, PVCL-Au or PVCL-MnO_2 NGs in the presence of X-ray irradiation (4 Gy).

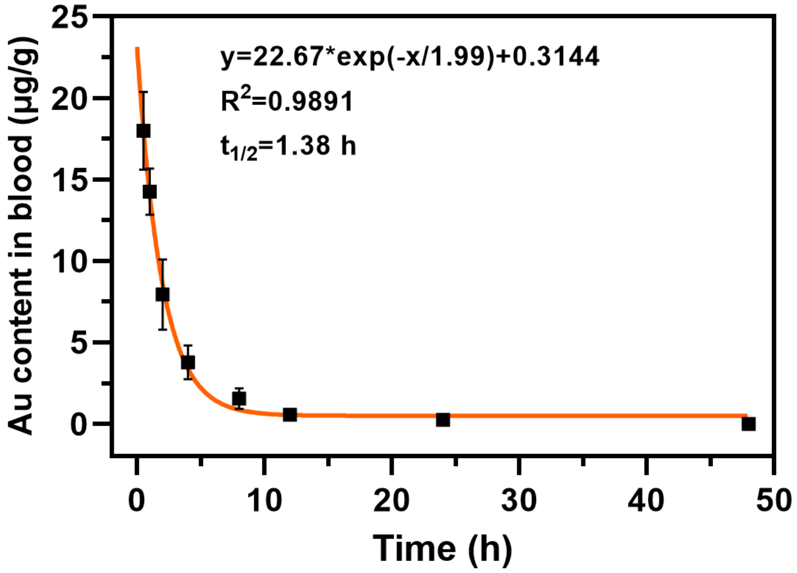

Figure S10. The pharmacokinetics of PVCL-Au-MnO_2 NGs after i.v. injection to mice (n = 3, [Au] = 10 mM, in 100 μL of PBS for each mouse).

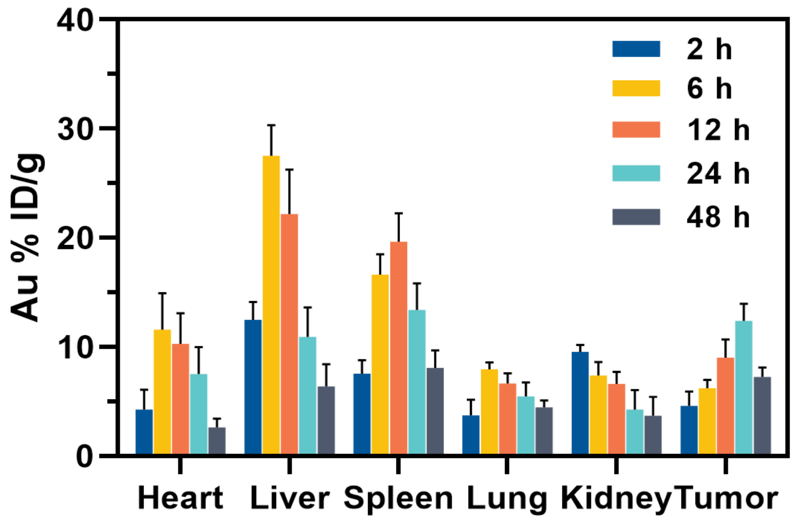

Figure S11. The biodistribution of Au (% injected dose (ID) of Au per gram of tissues) in main organs and tumors at 2, 6, 12, 24 and 48 h post i.v. injection of PVCL-Au-MnO_2 NGs (n = 3, [Au] = 10 mM, in 100 μL of PBS for each mouse).

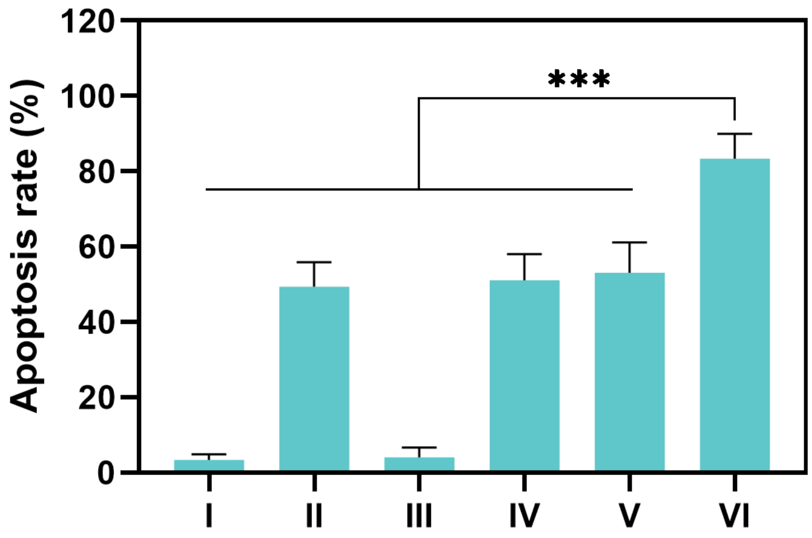

Figure S12. Quantitative analysis of the tumor cell apoptosis rate in various treatment groups (n = 5): (I) PBS, (II) X-ray (4 Gy), (III) PVCL NGs, (IV) PVCL NGs plus X-ray (4 Gy), (V) PVCL-Au-MnO_2 NGs, and (VI) PVCL-Au-MnO_2 NGs plus X-ray (4 Gy). *** represents $p < 0.001$.

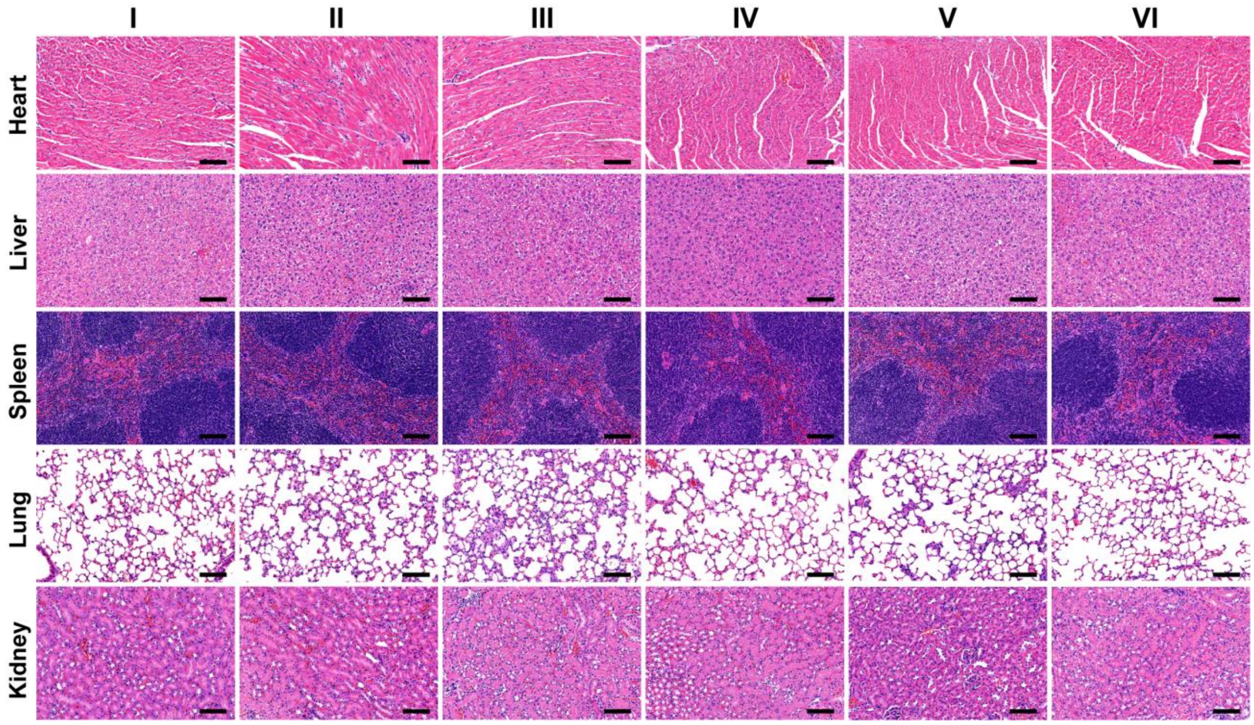

Figure S13. Representative H&E-stained images of major organs collected from tumor-bearing mice after different treatments for 20 days: (I) PBS, (II) X-ray (4 Gy), (III) PVCL NGs, (IV) PVCL NGs plus X-ray (4 Gy), (V) PVCL-Au-MnO_2 NGs, and (VI) PVCL-Au-MnO_2 NGs plus X-ray (4 Gy). Scale bar in each panel represents 200 μm.

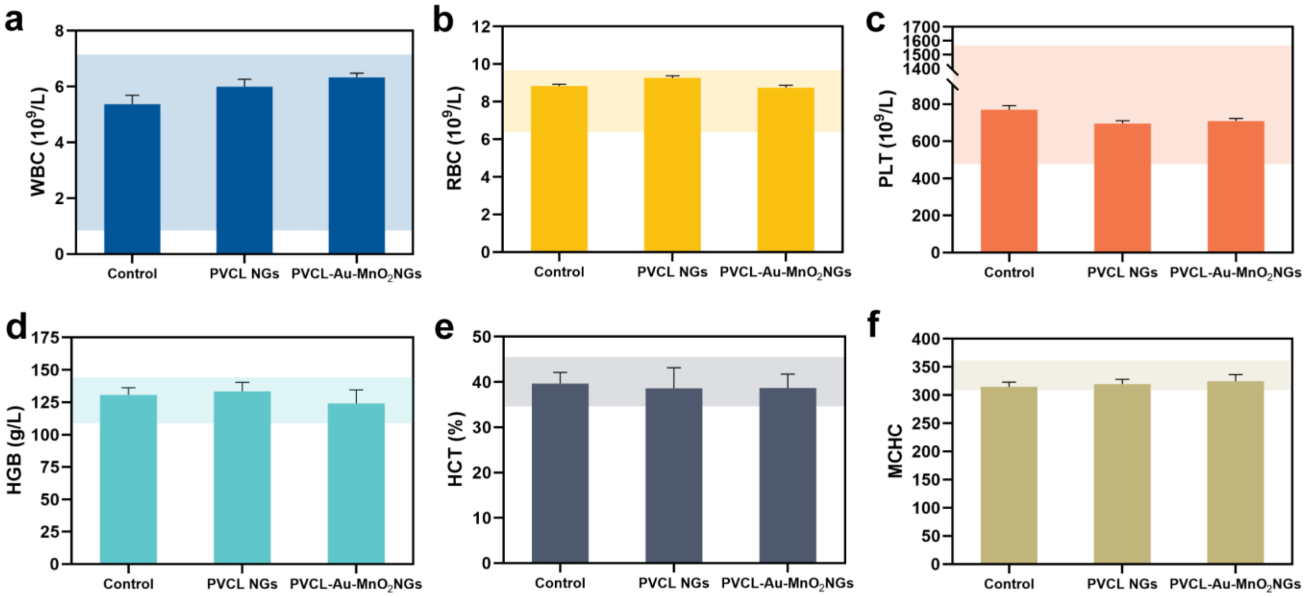

Figure S14. (a) WBC, (b) RBC, (c) PLT, (d) HGB, (e) HCT, and (f) MCHC of the mice at 7 days post i.v. injection of PBS, PVCL NGs and PVCL-Au-MnO_2 NGs, respectively. All data are shown as mean ± SD (n = 3). The shadow areas represent the referred normal ranges for healthy mice obtained from Servicebio, Inc. using Hematology Analyzer (Mindray, BC-2800 vet).

References

1. Lin L-S, Song J, Song L, Ke K, Liu Y, Zhou Z, *et al.* Simultaneous Fenton-like ion delivery and glutathione depletion by MnO_2-based nanoagent to enhance chemodynamic therapy. Angew Chem Int Ed Engl. 2018; 57: 4902-6.

2. Zhang C, Sun W, Wang Y, Xu F, Qu J, Xia J, *et al.* Gd-/CuS-loaded functional nanogels for MR/PA imaging-guided tumor-targeted photothermal therapy. ACS Appl Mater Interfaces. 2020; 12: 9107-17.

3. Fan Y, Tu W, Shen M, Chen X, Ning Y, Li J, *et al.* Targeted tumor hypoxia dual-mode CT/MR imaging and enhanced radiation therapy using dendrimer-based nanosensitizers. Adv Funct Mater. 2020; 30: 1909285.

4. Jiang W, Li Q, Xiao L, Dou J, Liu Y, Yu W, *et al.* Hierarchical multiplexing nanodroplets for imaging-guided cancer radiotherapy *via* DNA damage enhancement and concomitant DNA repair prevention. ACS Nano. 2018; 12: 5684-98.

5. Li J, Hu Y, Yang J, Wei P, Sun W, Shen M, *et al.* Hyaluronic acid-modified Fe_3O_4@Au core/shell nanostars for multimodal imaging and photothermal therapy of tumors. Biomaterials. 2015; 38: 10-21.
